# Genetic analysis of the *Candida albicans* biofilm transcription factor network using simple and complex haploinsufficiency

**DOI:** 10.1371/journal.pgen.1006948

**Published:** 2017-08-09

**Authors:** Virginia E. Glazier, Thomas Murante, Daniel Murante, Kristy Koselny, Yuan Liu, Dongyeop Kim, Hyun Koo, Damian J. Krysan

**Affiliations:** 1 Department of Pediatrics, University of Rochester Medical Center, Rochester, New York, United States of America; 2 Biofilm Research Laboratory, Levy Center for Oral Health, Department of Orthodontics, School of Dental Medicine, University of Pennsylvania, Philadelphia, Pennsylvania, United States of America; 3 Department of Microbiology and Immunology, University of Rochester Medical Center, Rochester, New York, United States of America; University College Dublin, IRELAND

## Abstract

Biofilm formation by *Candida albicans* is a key aspect of its pathobiology and is regulated by an integrated network of transcription factors (Bcr1, Brg1, Efg1, Ndt80, Rob1, and Tec1). To understand the details of how the transcription factors function together to regulate biofilm formation, we used a systematic genetic interaction approach based on generating all possible double heterozygous mutants of the network genes and quantitatively analyzing the genetic interactions between them. Overall, the network is highly susceptible to genetic perturbation with the six network heterozygous mutants all showing alterations in biofilm formation (haploinsufficiency). In addition, many double heterozygous mutants are as severely affected as homozygous deletions. As a result, the network shows properties of a highly interdependent ‘small-world’ network that is highly efficient but not robust. In addition, these genetic interaction data indicate that *TEC1* represents a network component whose expression is highly sensitive to small perturbations in the function of other networks TFs. We have also found that expression of *ROB1* is dependent on both auto-regulation and cooperative interactions with other network TFs. Finally, the heterozygous *NDT80* deletion mutant is hyperfilamentous under both biofilm and hyphae-inducing conditions in a *TEC1*-dependent manner. Taken together, genetic interaction analysis of this network has provided new insights into the functions of individual TFs as well as into the role of the overall network topology in its function.

## Author summary

Biofilm formation is part and parcel of the ability of *Candida albicans*, a normal component of the human gastrointestinal microbial community, to cause disease. Recent work by many investigators has provided detailed information regarding how *C*. *albicans* converts to the biofilm stage of growth. The vast majority of these studies have involved the study of strains lacking a single gene that affects biofilm formation. Using this genetic approach in combination with other genome-wide techniques, a network of transcription factors was identified that play a crucial role in regulating biofilm-related processes. Here, we have systematically generated and characterized strains with mutations in two biofilm network transcription factors in order to determine how these genes interact. This represents the first systematic quantitative genetic interaction study in *C*. *albicans* and, by combining our results with that of previous labs, provides a new level of detail regarding the integrated functions of these transcription factors. In addition, our data indicate that the biofilm transcription factor network is very sensitive to genetic perturbation and propose that this fragility is a function of its small-world topography which, in turn, promotes network efficiency at the expense of robustness.

## Introduction

*Candida albicans* is a fungal commensal of the human gastrointestinal tract with the potential to cause both superficial mucosal and life-threatening invasive infections [1]. As one of the most important human fungal pathogens, *C*. *albicans* has been the subject of intensive study and, accordingly, this work has informed our understanding of *C*. *albicans* pathobiology as well as fundamental paradigms of fungal infection biology [2]. This progress has been due, in large part, to the development and refinement of genetic tools applicable to *C*. *albicans* over the last twenty years [3]. As a result of the development of these technological and genetic resources, the roles of many important genes and pathways in *C*. *albicans* pathogenicity, host-response, and antifungal drug susceptibility have been delineated [4].

One genetic approach that has not been widely utilized in the study of *C*. *albicans* is genetic interaction or epistasis analysis. Large-scale genetic epistasis analysis was pioneered in the model organism *Saccharomyces cerevisiae* and has been extended to other organisms as well [5]. This powerful genetics approach allows the development of functional models and networks that inform our understanding of complex multi-genic phenotypes and processes [6]. One reason for the under-utilization of epistasis in *C*. *albicans* is the fact that it is, for the most part [7], a diploid organism without a standard meiosis-based mating process [8]. As a consequence, the high throughput, mating-based approaches to large-scale double mutant strain construction available in *S*. *cerevisiae* are not amenable to *C*. *albicans* genetics. Application of traditional genetic epistasis experiments to *C*. *albicans* requires the generation of double homozygous deletion mutant strains which is quite cumbersome, although recent developments in the application of CRISPR-Cas9 are likely to improve this process [9, 10]. Consequently, double homozygous deletion mutants have been used sporadically in the study of *C*. *albicans* and no systematic epistasis analysis using double homozygous mutants has been undertaken, to our knowledge.

As an alternative to epistasis analysis with double homozygous deletion mutants in *C*. *albicans*, we have adopted a genetic strategy referred to unlinked, non-complementation and initially applied to the study of essential genes in *S*. *cerevisiae* [11, 12]. More recently, it has been referred to as complex haploinsufficiency (CHI) because it involves the generation of strains that contain heterozygous deletion mutations at two separate loci. The double or complex heterozygote is then compared to each single heterozygote to determine if the double heterozygote phenotypes differ from that expected if the two single mutations affect the cell independently. It was adapted to systematic, genome-wide analysis by Haarer et al. in the context of generating the interaction network for the essential protein actin [12]. In *C*. *albicans*, we have used transposon-based mutagenesis approaches to study the CHI-interactions of the **R**egulation of **A**ce2 and **M**orphogenesis (RAM) pathway [13, 14]. CHI-based analysis has revealed the functional interaction between the PKA and RAM pathways and has provided insights into the wide-range of processes related to the RAM pathway. However, prior to the work reported here, a systematic genetic interaction analysis has not been reported for *C*. *albicans*, to our knowledge.

Systematic genetic screens using ordered collections of homozygous deletion mutants in *C*. *albicans* have identified large sets of genes that are required for a variety of phenotypes including filamentation, biofilm formation, and drug susceptibility [15, 16, 17]. These important studies, as well as single-gene investigations, have provided a portrait of the individual genes that are involved in a variety of phenotypes with relevance to *C*. *albicans* biology and pathobiology. A landmark example of this approach is the work of Nobile et al. who extensively characterized a six transcription factor (TF) network that regulates biofilm formation [18]. The network is comprised of the following transcription factors: Brg1, Ndt80, Rob1, Tec1, Bcr1 and Efg1. Of the target genes regulated by these TFs, almost half were bound by two or more regulators. These data indicate that multiple transcriptional regulators regulate the expression of key biofilm effector genes [18]. Nobile et al. also demonstrated that each TF regulates its own expression as well the expression of at least one other network TF [18]. Taken together, these data indicate that the transcriptional circuitry for biofilm formation is highly integrated. However, the functional consequences of this integration and the identities of pairs of TFs that functionally interact are not yet known. Our approach was designed to begin to address this question.

An important characteristic of the biofilm TF network identified by Nobile et al. is that the homozygous deletion mutants of any of the six core TFs leads to a profound reduction in biofilm formation [18]. Therefore, genetic interaction analysis using double homozygous deletion mutants will be limited because it will be difficult to detect additional changes in biofilm formation relative to either single homozygous strain. Consequently, we hypothesized that CHI-based epistasis analysis would circumvent this limitation and provide further insights into how they function together to regulate this integral part of *C*. *albicans* infection biology.

Here, we describe an analysis of the biofilm TF network using both simple and complex haploinsufficiency. We found that the network TFs all show haploinsufficiency with respect to either biofilm density or architecture. In addition, we have found that many double heterozygous strains have defects in biofilm density that are comparable or more severe than homozygous deletions. These observations, along with the decreased biofilm formation in the homozygous deletions, indicate that the biofilm TF network is not genetically robust but rather is quite fragile. The topological structure of the TF network is consistent with a small-world model based on its high coefficient of connectivity and very short average path length between any two nodes. Such networks rapidly transmit information throughout the network and, thus, are highly efficient. The topological structure of the network, therefore, promotes efficiency at the expense of genetic robustness. Finally, analysis of individual double heterozygous mutants has provided new insights in to how some of the TFs function together to regulate each other’s expression and to mediate compensatory responses. Taken together, the systematic analysis of the biofilm TF network using simple and complex haploinsufficiency has provided an additional level of resolution regarding its structure and function.

## Results

### Deletion mutants of biofilm network TFs display simple and complex haploinsufficiency

**Fig 1:**
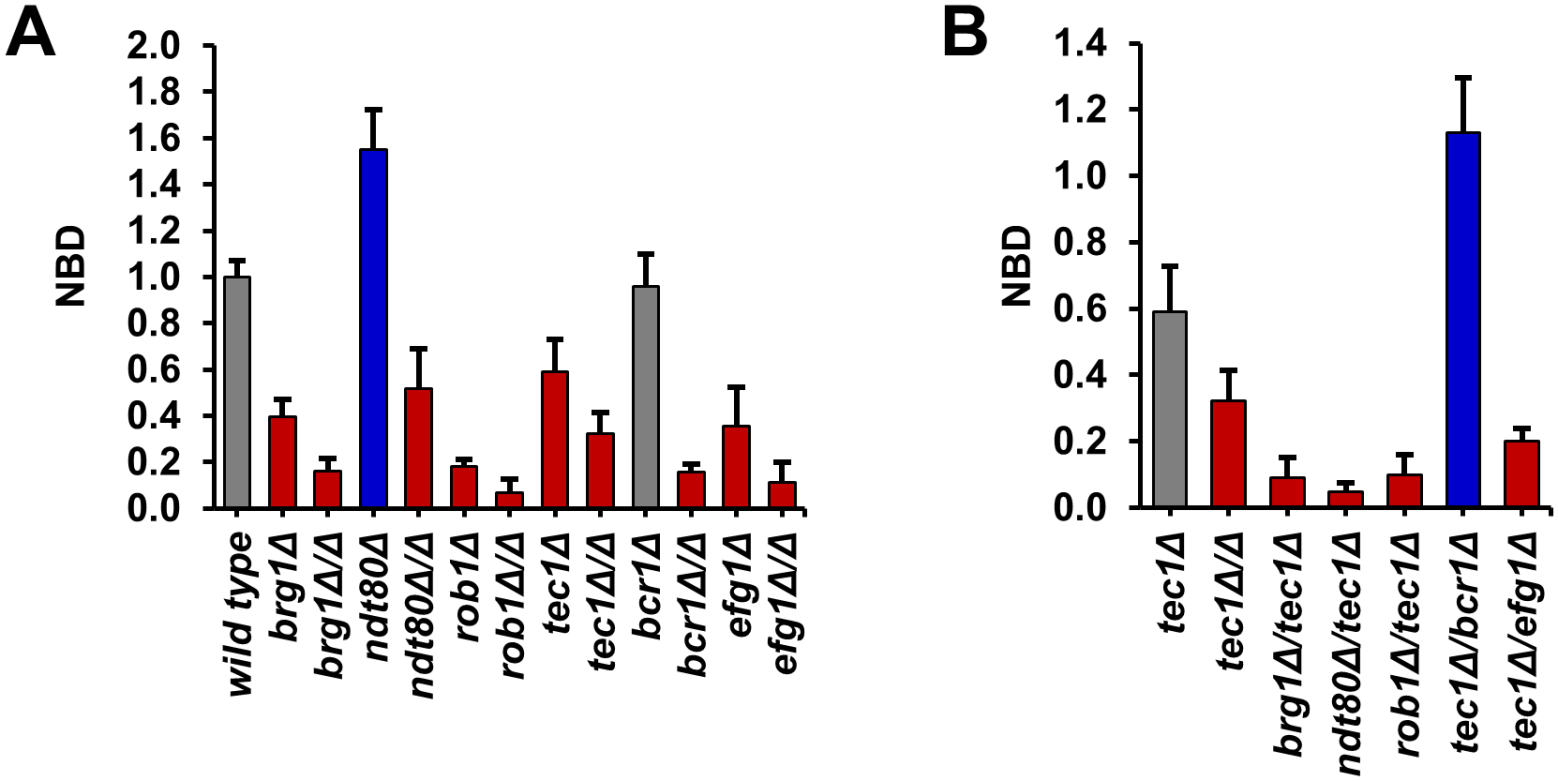
Heterozygous deletion mutants of six core biofilm transcription factors show simple and complex hapoinsufficiency for biofilm density. (A) The density of the biofilms formed by the indicated wild type (SN250), heterozygous and homozygous deletion strains was determined by measuring the OD_600_ after incubation for 48 hr as described in Materials and Methods (YETS medium). The raw data were normalized to wild type: Normalized Biofilm Density (NBD) = OD_600_ mutant/OD_600_ wild type. Data are representative of results obtained on at least three different days. Bars are the mean of three or four independent well replicates. Error bars are standard deviation. Red and blue bars indicate statistically significant reduction and increase from wild type, respectively (Student’s t test, *P*<0.05). Grey bars indicate no change. (B) The interaction profile for the interactions of *tec1*Δ/*TEC1* with all five other network TFs is shown. Bars are the mean of three or four independent well replicates. Error bars are standard deviation. Red and blue bars indicate statistically significant reduction and increase from the *tec1*Δ/*TEC1* heterozygote, respectively (Student’s t test, *P*<0.05). Grey bars indicate no change.

To investigate the genetic interactions within the biofilm TF network, we constructed a set of deletion mutants that contained heterozygous deletion mutants of each TF and double heterozygous mutants corresponding to all possible combinations of the heterozygous deletions (total of 15 combinations = 6!/2!(4!)). The strains were constructed by amplifying deletion cassettes from homozygous deletion mutants contained within the collection deposited by Homann et al. [16] at the Fungal Genetics Stock Center to yield *LEU2*-marked cassettes while the *HIS1*-derived constructs were generated using a *HIS1* plasmid template and standard techniques. The heterozygous nature of the mutants was confirmed by quantitative DNA PCR and all showed the expected reduction in copy number relative to the parental reference strain. The effect of each mutation on in vitro biofilm formation as well as on the expression of the each TF in the network was determined. The genotypes for all strains are provided in S1 Table and the normalized biofilm density data for all double heterozygotes is provided in (Fig 1B and S1 Fig). The effect of each TF single and double heterozygous mutation on the expression of other network TFs is provided in S2 Table.

A number of different assays have been applied to the assessment of in vitro biofilm formation by *C*. *albicans* and their relative advantages and limitations have been recently examined and reviewed [19, 20]. Biofilms are complex biological structures and no single assay can measure all of its features. Our goal was to identify a highly reproducible assay that provided a general measure of biofilm formation. Based on previous literature reports and our own pilot studies, we selected the optical density assay described by Fox et al. for the analysis of the same TF network as we are studying in this work [21]. In this assay, *C*. *albicans* strains are incubated in a microtiter plate with a given medium to generate an adherent biofilm which is then carefully washed to remove suspended cells. The OD_600_ of the well is then measured [20,21].

As recently described in their assessment of the different methods to measure *C*. *albicans* biofilm formation, Lohse et al. found that the optical density assay has the advantage of minimal processing and no reagent-based issues [20]. As Lohse et al. also note, the optical density assay is a direct measurement of biofilm formation and is not affected by penetration of reagents or by the metabolic activity of the different mutants [20]. As detailed below, representative mutants with medium and severe defects by the optical density assay also had similar defects by confocal microscopy. One of the limitations of the assay is that it does not provide structural information and, thus, mutants that form biofilms with the same optical density may have structural differences or different ratios between cellular density and extracellular matrix. For the purposes of our large scale genetic analysis, we felt the optical density assay provided a good balance between sensitivity and specificity.

We limited our analysis to biofilm density at 48 hr. In addition to the heterozygous and double heterozygous mutants described above, we also determined the density of biofilms formed by the corresponding homozygous deletion mutants. Consistent with the data reported by Nobile et al. [18] and Fox et al. [21], all six of the homozygous TF deletion strains showed statistically significant reductions in biofilm density (P < 0.05, Student’s t test, Fig 1A)]. In addition, the OD_600_ readings for WT in YETS at 48 hr were essentially identical to those observed by Fox et al. for biofilm formation in Spider medium [21]. The heterozygous TF mutants were also tested under the same conditions (Fig 1A). Four out of the six TFs (*BRG1*, *EFG1*, *ROB1*, and *TEC1*) showed haploinsufficiency relative to the WT reference strain. Furthermore, the four strains showing haploinsufficiency formed biofilms that were less dense than WT but more dense than the corresponding homozygous deletion strain.

The *BCR1* heterozygous deletion mutant showed no difference in biofilm density relative to WT, while the *NDT80* heterozygote has increased biofilm density. Interestingly, the homozygous deletion strains of both *BCR1* and *NDT80* are, as previously reported [18], severely deficient for biofilm formation. Thus, five out of the six TF heterozygotes show phenotypes based on optical density measurements. Previous genome-wide studies of haploinsufficiency in the model yeast *S*. *cerevisiae* indicate that only ~3% of genes are haploinsufficient [22]. The elevated frequency of haploinsufficient/-proficient phenotypes in this set of TFs is consistent with previous studies indicating that highly integrated sets of genes such as protein complexes are much more likely to show haploinsufficiency [23].

Ten of the fifteen double heterozygous deletion mutants showed reduced biofilm density relative to both of the single mutants, indicating deleterious complex haploinsufficiency. Furthermore, a significant number of the double heterozygote mutants are as deficient in biofilm formation as homozygous mutants: specifically all *BRG1* pairs; 4 *TEC1* pairs; 3 *EFG1* pairs; 3 *NDT80* pairs; 2 *ROB1* pairs; and 2 *BCR1* pairs are similar to, or less fit than, the homozygous deletion of one of the pairs in the double mutant. A specific example in the form of the set of *tec1*Δ/*TEC1* derivatives is provided in Fig 1B; similar graphs for the remaining five combinations are provided in S1 Fig. The double heterozygotes of all but one of the pairs involving *TEC1* have less dense biofilms than the *tec1*Δ/Δ. Thus, loss of two alleles at distinct loci leads to phenotypes that are similar to loss of both alleles at one loci. These observations further support the highly integrated nature of the biofilm TF network. In addition, these data indicate that the network is exquisitely sensitive to the gene dosage.

### Biofilm TF heterozygotes have altered expression of network TF genes

**Fig 2:**
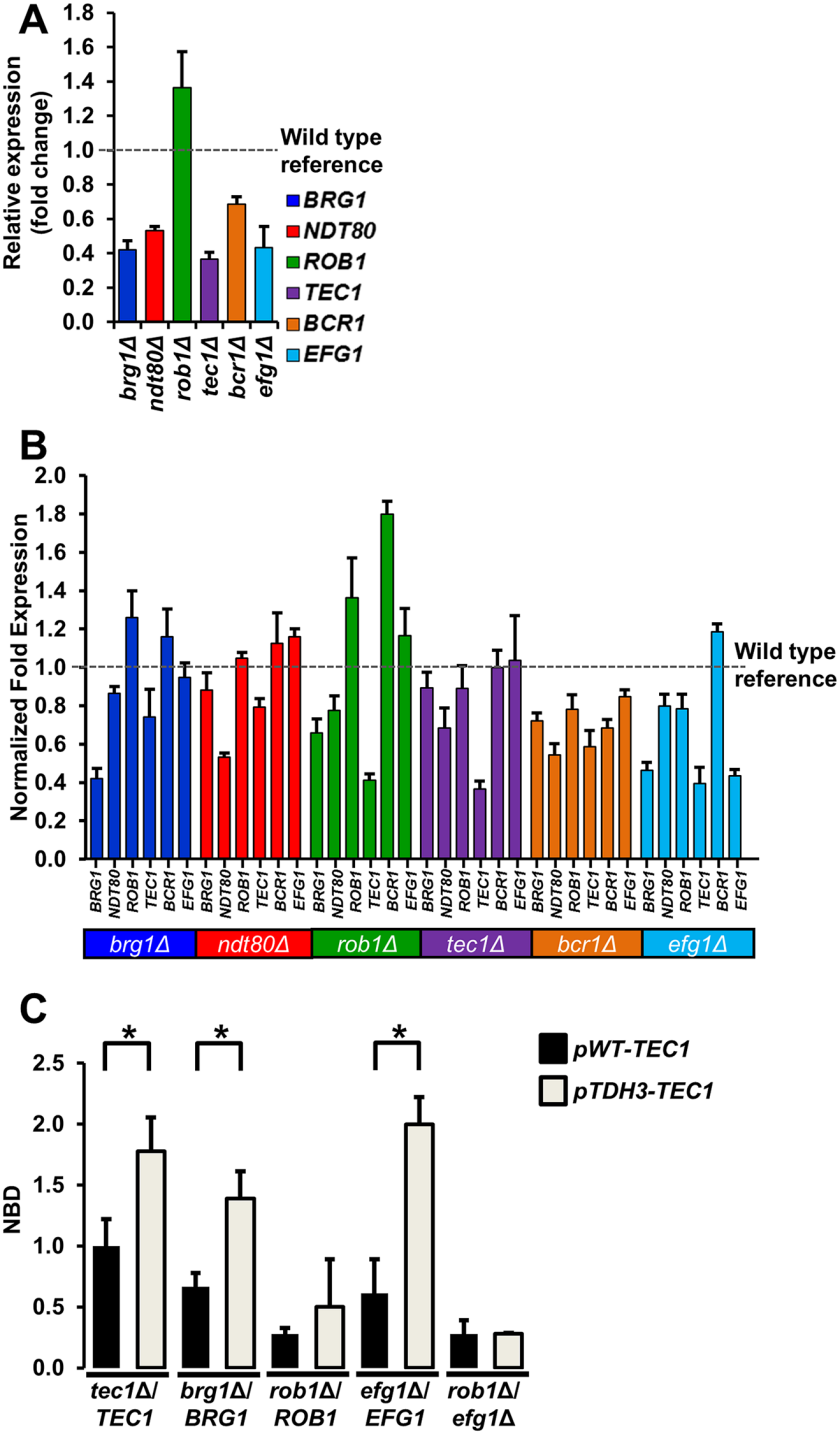
Heterozygous biofilm trancription factor mutants show haploinsufficient expression of network transcription factors. (A) The expression of each of the six biofilm TFs (*BRG1*, *BCR1*, *EGF1*, *NDT80*, *ROB1*, and *TEC1*) was measured by qRT-PCR in the corresponding heterozygous deletion mutant of that TF after 48hr biofilm formation and expressed relative to WT (SN250). The bars indicate mean relative expression for three or four independent experiments performed in triplicate and error bars are SEM. (B) The expression of each of the six core biofilm TF was determined for all six of the heterozygous TF deletion mutants after 48 hr biofilm conditions. The expression levels for each gene are normalized to WT (SN250) under the same conditions. Each bar represents the mean of three independent experiments performed in triplicate. The error bars indicate SEM. (C) The normalized biofilm densities (NBD), OD_600_ mutant/OD_600_ *tec1*Δ/*TEC1* *pWT-TEC1*, at 48hr are shown for the indicated strains. Strains with the *pWT-TEC1* designation have both *TEC1* alleles expressed from the endogenous promoter. Strains with the in *pTDH3-TEC1* designation contain one allele of *TEC1* regulaed by the *TDH3* promoter. Data are representative of results obtained on at least three different days. Bars are the mean of three or four independent well replicates. Error bars are standard deviation. Brackets with asterisks indicate a statistically significant difference between the two means (*P*<0.05, Student’s t test). Absence of a bracket and asterisk indicates that there is no significant difference between means.

The most well-accepted and experimentally-supported mechanism for haploinsufficiency is that a reduction in gene copy leads to a corresponding reduction in gene expression [22, 23]. Springer et al. showed that deletion of one allele in *S*. *cerevisiae* results in a two-fold reduction in the expression of that gene for >80% of the genes examined [24]. In *C*. *albicans*, Uhl et al. noted similar finding in the context of a large-scale haploinsufficiency screen for genes affecting filamentation [25]. As shown in Fig 2A, four (*TEC1*, *EFG1*, *BRG1*, *NDT80*) of the six biofilm TF displayed ~2-fold reduction in gene expression. Despite its apparent haploproficient phenotype, the *NDT80* heterozygote has almost exactly one-half the expression of its gene product relative to wild type. Thus, the haploproficient phenotype is not due to a compensatory process that maintains expression of *NDT80* but appears instead to involve indirect responses to the reduction in *NDT80* gene dosage. As described below, we have used CHI analysis to identify TFs mediating this response.

In distinct contrast, *ROB1* RNA levels are increased in the *rob1*Δ/*ROB1* strain compared to wild type. Allelic differences in the promoter region may result in preferential expression of one allele [26]. However, previous transcriptional and translational profiling of the *ROB1* alleles did not reveal an allelic bias under normal growth conditions [26]. Thus, the most likely explanation for this observation is that it represents an example of dosage compensation; Springer et al. found that this occurs in less than five percent of genes [24]. The observed dosage compensation for *ROB1* suggests that the non-linear relationship between the phenotypes of the homozygous and heterozygous mutants is not related to the expression of levels of *ROB1*. As detailed below, genetic interaction analysis allowed us to determine that *ROB1* expression is highly dependent on both auto-regulation and cooperative interactions with other network TFs.

We also measured the effect of TF gene dosage on the expression of the other five network TFs (Fig 2B). Only two TFs showed significant reductions in expression in strains other than their own heterozygotes; *BRG1* and *TEC1*. *BRG1* expression was reduced two-fold in an *efg1*Δ/*EFG1* heterozygote. Most strikingly, *TEC1* expression was reduced by at least 1.5-fold in three of the remaining five heterozygotes: *EFG1*, *BCR1*, and *ROB1*. This suggested that *TEC1* expression might be an important network output. If this were the case, then expression of *TEC1* in a manner independent of the TF network could be expected to rescue the haploinsufficiency of other TF heterozygotes. We, therefore, inserted the strong, constitutive *TDH3* promoter (p*TDH3*) upstream of *TEC1* in five heterozygous or double heterozygous TF mutants; we did not construct the corresponding *bcr1*Δ/*BCR1* derivative because Tec1 has already been shown to regulate *BCR1* expression [27] and the heterozygote does not show haploinsufficiency by optical density (Fig 1A). The *TDH3* promoter has been used previously to induce overexpression in the context of biofilm formation [27, 28].

Overexpression of *TEC1* in WT increased biofilm density modestly (1.66 to 1.94 NBD compared to *tec1*Δ/*TEC1*) and complemented the haploinsufficient phenotype of *tec1*Δ/*TEC1* (Fig 2C). Similarly, placement of *TEC1* under the control of the p*TDH3* increased biofilm density of the *brg1*Δ/*BRG1* and *efg1*Δ/*EFG1* strains. Indeed, increased expression of *TEC1* restored the biofilm density of the *efg1*Δ/*EFG1* mutant to near wild type levels (Fig 2C). In contrast, the biofilm density of the *rob1*Δ/*ROB1* strain was essentially unaffected by p*TDH3-TEC1* relative to its parental strain. Based on these observations, we suspected that the ability of p*TDH3*-driven *TEC1* expression to modulate biofilm density of haploinsufficient TF heterozygotes may be dependent upon Rob1. Consistent with that model, p*TDH3-TEC1* had no effect on the biofilm density of the *rob1*Δ/*ROB1 efg1*Δ/*EFG1* mutant (Fig 2C). These data indicate that at least some of the functions of Tec1 are dependent on Rob1 and are consistent with the possibility that these two TFs function in a linear pathway. Furthermore, it appears that reduced expression of *TEC1* plays an important role in the haploinsufficiency of *EFG1*.

### Analysis of TF expression in biofilm TF double heterozygotes identifies regulators of *ROB1* expression

**Fig 3:**
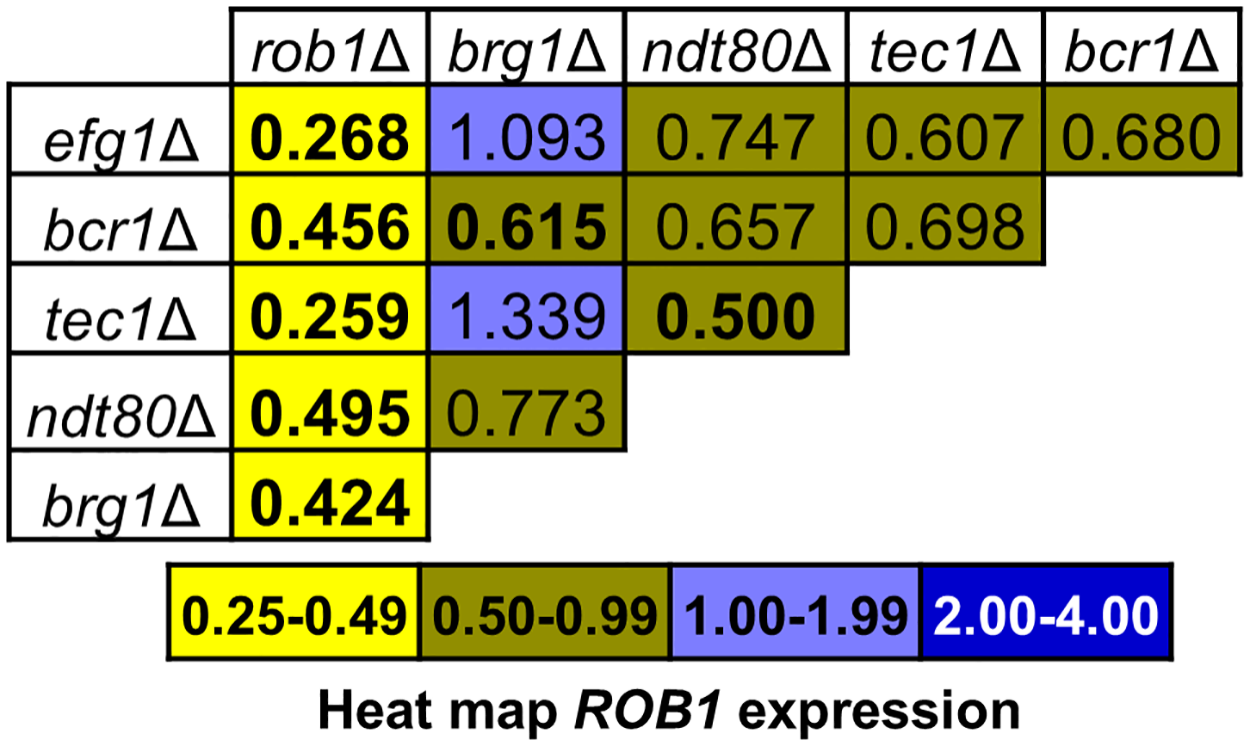
*ROB1* expression is dependent on auto-regulation and cooperative interactions with other network transcription factors. The heat map shows the fold change in *ROB1* expression of each double mutant relative to WT. The data for each of the corresponding single mutants is shown in Fig 2 and the raw data for all strains is provided in S2 Table. None of the single heterozygous strains showed a statistically signficant effect on *ROB1* expression. Bold indicates that the fold change from the two single heterozygotes is statistically significant (*P*< 0.05, Student’s t test).

The expression data for the homozygous biofilm TF deletion mutants reported by Nobile et al. [18] did not identify any TFs that regulated *ROB1* or *EFG1* [18]. The expression levels for the other four TFs were reduced in at least one other network TF homozygous deletion mutant. We, therefore, used our set of double heterozygous mutants to determine if combinations of deletions mutants led to reduced expression of these two TFs. The heat map for *ROB1* expression for the full set of biofilm TF mutants is shown in Fig 3. *ROB1* expression is not reduced in the heterozygous *rob1*Δ/*ROB1* mutant (Fig 2A), indicating that dosage compensation may be operative [24]. Deletion of one allele of any network TF when paired with the *ROB1* heterozygote reduced *ROB1* expression relative to the *ROB1* single heterozygote by at least 2-fold. Three TFs has been shown by Nobile et al. to bind the promoter of *ROB1*: Tec1, Efg1, and Ndt80. Although the *bcr1*Δ/*BCR1* and *brg1*Δ/*BRG1* pairs show reduced *ROB1* expression, neither TF directly binds to the promoter of *ROB1* [18] and, therefore, Bcr1 and Brg1 appear to play indirect roles in *ROB1* expression (Fig 3 and S2 Table). The double mutant of *TEC1* and *NDT80* shows reduced expression suggesting that, of the three directly binding TFs involved in *ROB1* expression, they may be the most important. Based on single gene deletion studies, the binding of Tec1, Efg1, and Ndt80 to the promoter of *ROB1* represents an unproductive binding event in that they bind to the promoter of *ROB1* but do not apparently affect *ROB1* expression [18]; however, our complex haploinsufficiency study reveals that these binding events are productive but are dependent on the auto-regulatory function of Rob1 with respect to its own expression.

Interestingly, the expression of *TEC1*, *NDT80* and *EFG1* are all reduced by 1.5–3.0-fold in the *bcr1*Δ/*BCR1 brg1*Δ/*BRG1* mutant (S2 Table). From these genetic interactions, the indirect and direct regulation of *ROB1* expression by the TF network can be summarized as follows. Bcr1 and Brg1 play indirect roles which appear to be due to the fact that both TFs are required for full expression of the direct regulators of *ROB1* expression. Ndt80, Tec1, and Efg1 all directly bind the promoter of *ROB1* and, therefore, directly contribute to its expression in an overlapping or redundant manner since homozygous deletion of anyone of the TFs does not affect *ROB1* expression. *ROB1* expression represents a positive feed-back loop because Rob1 is crucial for its own expression. However, this auto-regulatory process also requires each of the direct regulators for wild type levels of expression because reduction in gene copy of anyone in the *ROB1* heterozygous mutant leads to dramatically reduced expression. Reduction in *ROB1* gene copy does not affect overall expression as long as wild type levels of each of the direct regulators are present.

Like *ROB1*, none of the homozygous deletion mutants of the network TFs has an effect on *EFG1* expression [18]. Consistent with that observation, only the *efg1*Δ/*EFG1* heterozygote showed reduced *EFG1* expression (Fig 2B). All six TFs bind to the promoter of *EFG1* suggesting that multiple TFs are likely to make redundant contributions to its expression [18]. However, unlike *ROB1* expression, none of the double mutants, including those pairing *EFG1* with the other TFs, showed reduced expression relative to the *efg1*Δ/*EFG1* heterozygote. Thus, it appears that either more than two TFs make redundant contributions to *EFG1* expression or its expression is regulated by one or more extra-network TF(s).

### *NDT80* heterozygote forms dense biofilms with altered cellular structure in a Tec1 and Rob1 dependent manner

**Fig 4:**
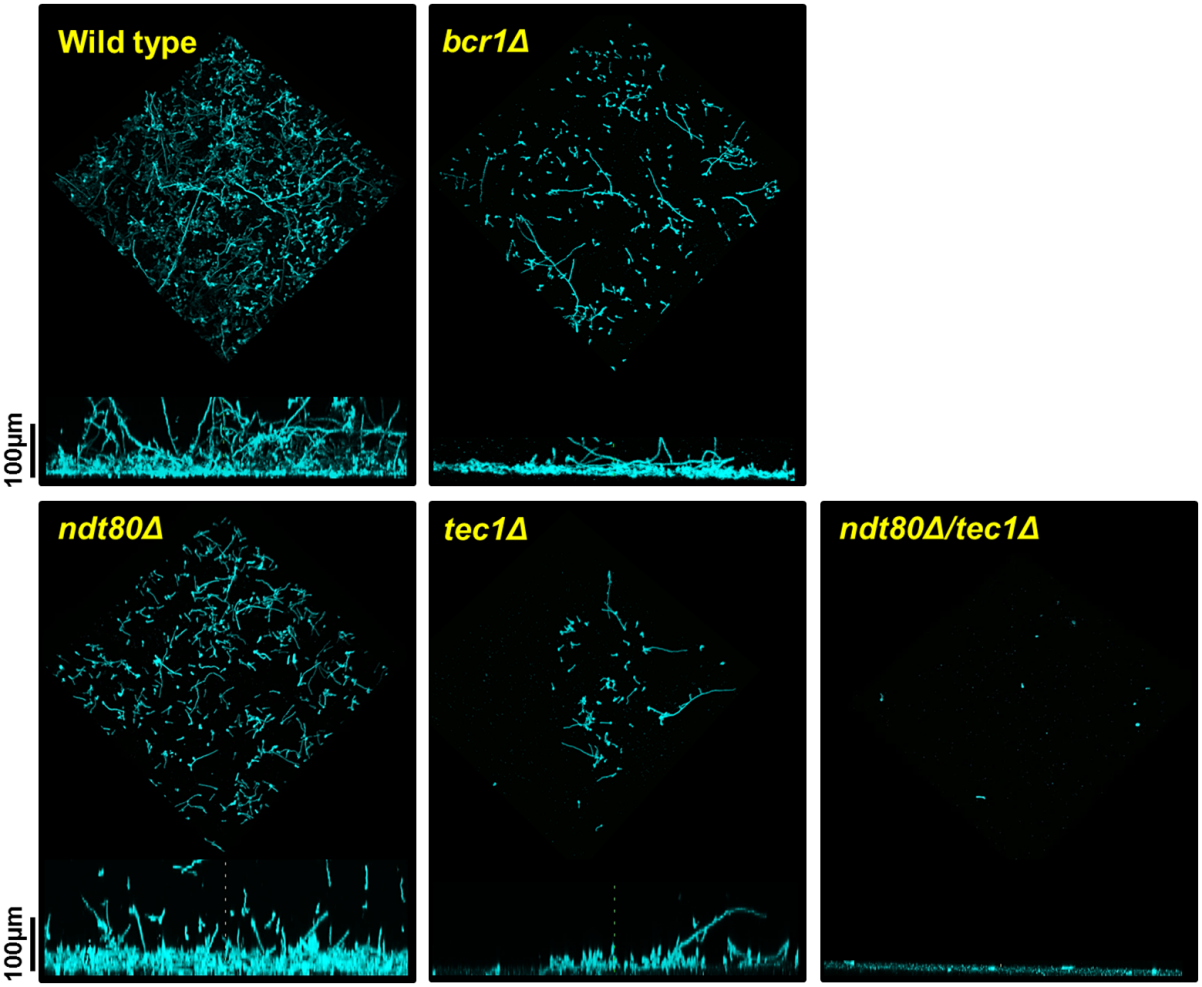
The *ndt80*Δ/*NDT80* and *bcr1*Δ/*BCR1* show altered biofilm architecture. Confocal microscopy images of wild type, *ndt80Δ/NDT80*, *bcr1*Δ*/BCR1*, *tec1Δ/TEC1*, and *ndt80Δ/NDT80 tec1Δ/TEC1* strains grown in YETS for 48 hr. Cells were stained with concanavalin A. See Materials and methods for full details. The top image is a three-dimensional rendering of the biofilm. The bottom image is a cross-sectional view of the same biofilm.

As discussed above, the biofilm density assay does not distinguish between two strains that have similarly dense biofilms but may be have structurally distinctions or altered amounts of extracellular matrix. This distinction is particularly relevant for deletion strains that show no apparent phenotypes relative to WT. We, therefore, examined the biofilms formed by the set of heterozygotes as well as one double heterozygous mutant. Representative examples of these data are shown in Fig 4 and include strains with biofilm densities similar to wild type (*bcr1*Δ*/BCR1*); increased relative to WT (*ndt80*Δ*/NDT80)*; mildly decreased from WT (*tec1*Δ/*TEC1*); and severely decreased relative to WT (*ndt80*Δ*/NDT80 tec1*Δ/*TEC1*). The remaining heterozygous strains are shown in S2 Fig. As shown in Fig 4, the strains with either increased or normal biofilm densities both showed architectural changes relative to WT. The *bcr1*Δ*/BCR1* strain has reduced cellularity relative to WT; by this, we mean the number of distinct cells appears lower. This discrepancy has two possible explanations. First, it is possible that the biofilm is more fragile and, therefore, less stable to the processing required for confocal microscopy. Second, the *bcr1*Δ*/BCR1* mutant may produce a biofilm that has increased amounts of extracellular matrix which masks the reduction in cellular contribution to the biofilm mass, a feature that is best assessed by biochemical methods. The *ndt80*Δ*/NDT80* strain has increased density along the basal layer relative to WT. The *tec1*Δ/*TEC1* and *ndt80*Δ*/NDT80 tec1*Δ/*TEC1* strains show reductions in cellularity and biofilm thickness that closely follow the trends of the biofilm density measurements.

**Fig 5:**
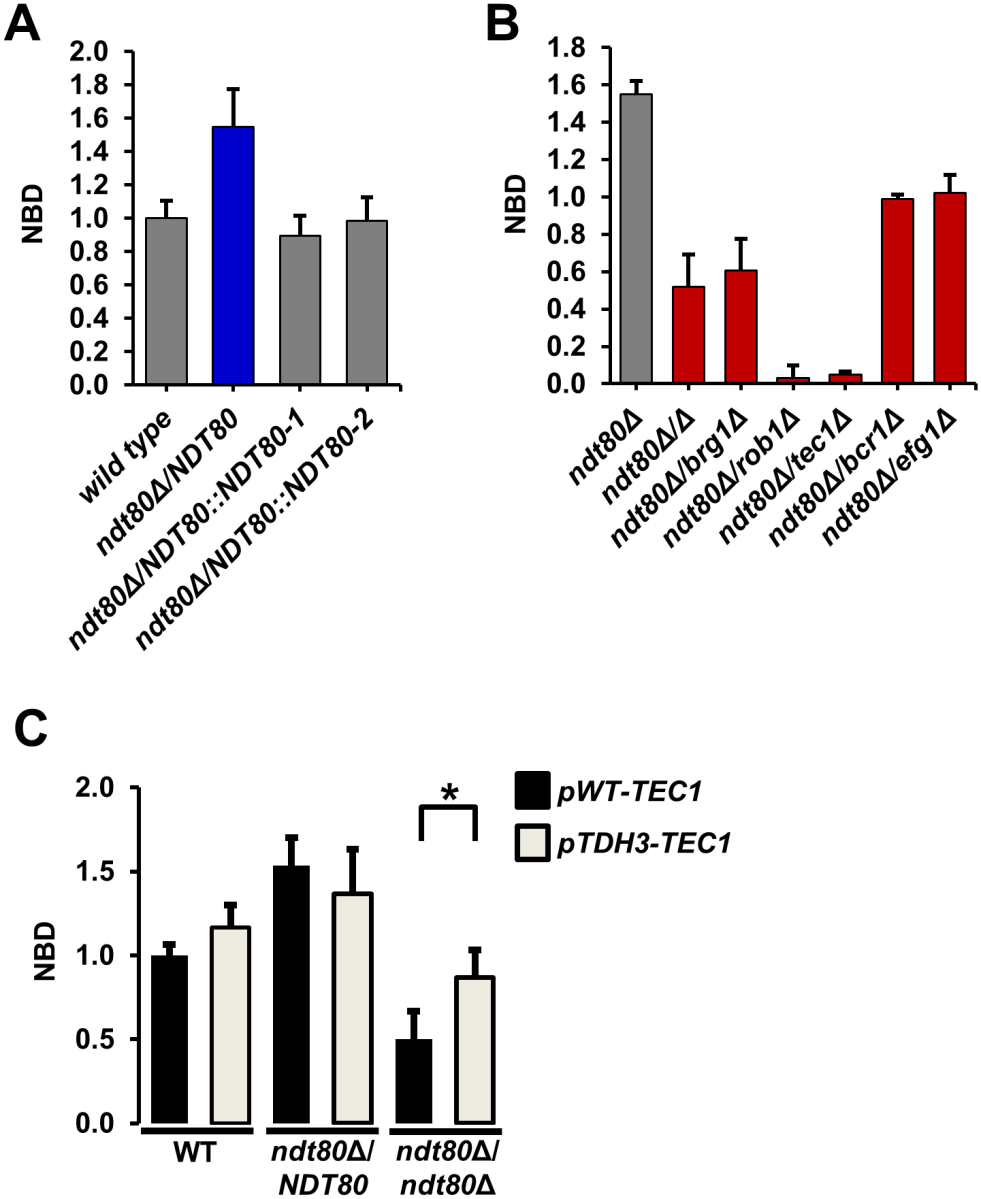
The altered architecture of *ndt80*Δ/*NDT80* is strongly dependent on Rob1 and Tec1. (A) Complementation of *ndt80*Δ/*NDT80* with an allele under endogenous promoter restores wild type levels of normalized biofilm density (NBD) NBD=OD_600_ mutant/OD_600_ wild type. The blue bar indicates statistically significant increase relative to WT. Grey bars indicate no difference from WT. (B) The increased biofilm density of *ndt80*Δ/*NDT80* is dependent on other network TFs with Tec1 and Rob1 having the most dramatic effects. The red bars indicate statistically significant reduction relative to WT and *ndt80*Δ/*NDT80* density. (C) Overexpression of *TEC1* with the strong constitutive promoter *TDH3* partially restores biofilm formation to the *ndt80*Δ/Δ mutant. Asterisk indicates statistically significant difference between means for strains with and without p*TDH3-TEC1* allele as determined by Student’s t test with significance level set at *P*<0.05.

We were interested in probing the basis of the altered architecture and increased density phenotype of the *ndt80*Δ*/NDT80* because it is quite distinct from the phenotype showed by the *ndt80*Δ/Δ homozygous deletion mutant. The *ndt80*Δ/Δ mutant has a significant defect in biofilm density as shown in Fig 1A. First, we confirmed that the increased density was dependent on loss of one copy of *NDT80*. Re-introduction of an *NDT80* allele under its endogenous promoter restored the biofilm density to WT levels (Fig 5A), confirming the gene dosage dependence of the *ndt80*Δ*/NDT80* phenotype. Second, we examined the full set of *ndt80*Δ/*NDT80* double heterozygotes to identify other TFs that may be required for the phenotype (Fig 5B). Although the density of the biofilms was reduced in each of the double mutants relative to the parental, deletion of one allele of *TEC1* and *ROB1* in the *ndt80*Δ*/NDT80* background had profound effect on biofilm density and, in the case of *TEC1*, architecture (Fig 4). Because *TEC1* and *ROB1* expression is maintained near wild type levels in *NDT80* (S2 Table), it would appear that the increased biofilm density is dependent on these two TFs and that the *TEC1-ROB1* pair may mediate a compensatory response. Furthermore, *TEC1* expression is reduced by >10-fold in the *ndt80*Δ*/NDT80 tec1*Δ/*TEC1* mutant (S2 Table). We, therefore, hypothesized that *TEC1* may play an important role in a compensatory response to reduced *NDT80* gene dosage. To test this, we placed *TEC1* under the control of *TDH1* in the *NDT80* deletion strains (Fig 5C). This did not have a statistically significant effect on the *NDT80* heterozygote but the biofilm density of the *ndt80*Δ/Δ was increased significantly. As we have shown above, the ability of *TEC1* to suppress the biofilm defects of other network TFs is dependent on *ROB1*. Taken together, these genetic interaction and over-expression data are consistent with a model in which the increased density of the *NDT80* heterozygote is dependent on a pathway involving *TEC1* and, possibly, *ROB1*.

**Fig 6:**
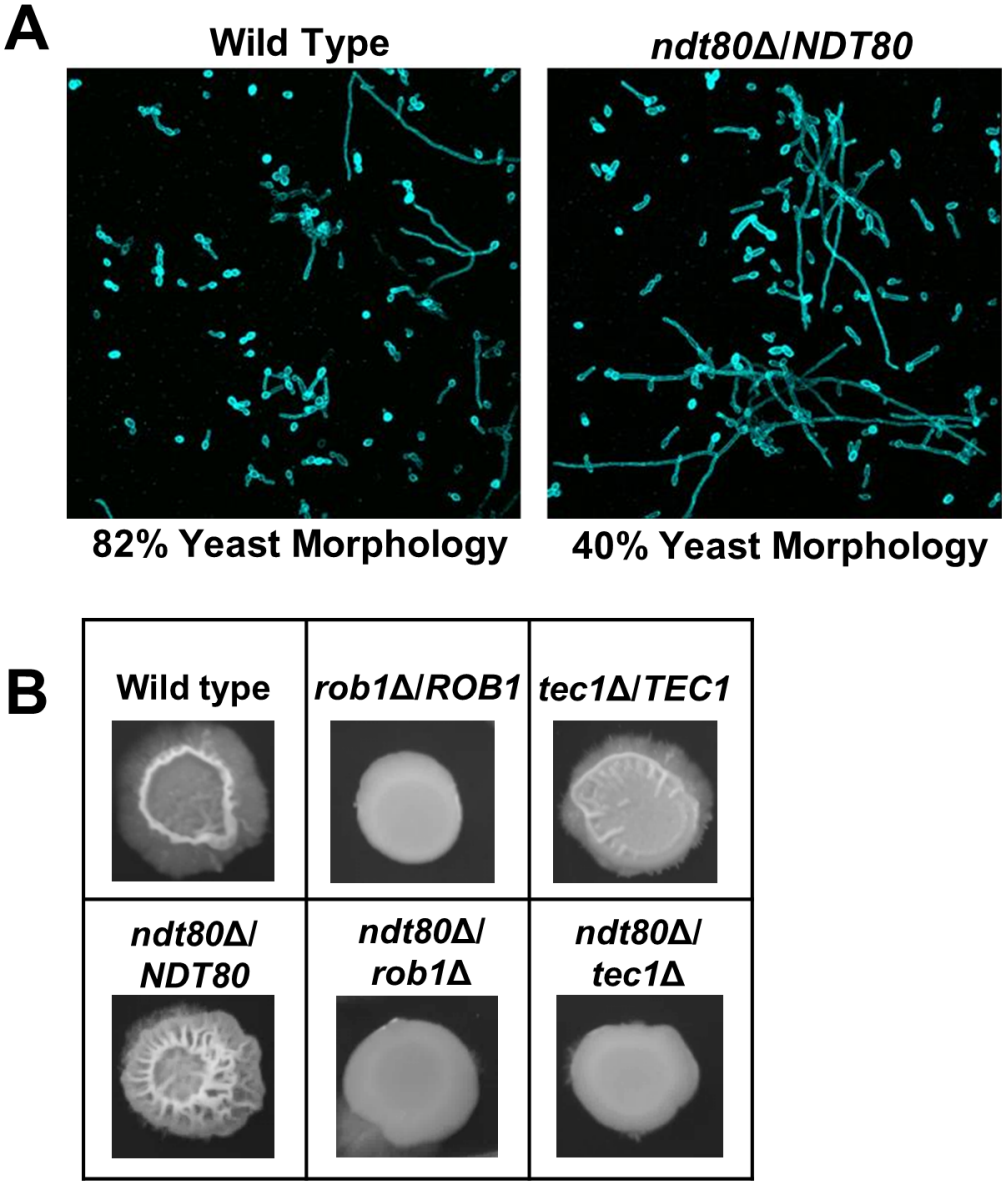
The basal layer of the *ndt80*Δ/*NDT80* biofilm has an increasaed proportion of filaments relative to WT. (A) High magnification view of biofilm centered at 30 nm from solid surface. The relative proportion of yeast and filamentous cells (hyphae+pseudohyphae) was based on counting at least 100 total cells for the two strains. (B) The indicated strains were spotted on Spider medium and incubated at 37°C before the photographs were taken.

To further explore the nature of the increased biofilm density of *ndt80*Δ/*NDT80*, we examined the cells at the basal layer of the biofilm. In a cross-section view of the basal layer of the biofilm, the *ndt80*Δ/*NDT80* cells appear more filamentous than the WT strain (Fig 4). Consistent with this assessment, the coronal views of that layer indicate that the number of cells with a yeast morphotypes increased by 2-fold in WT is in comparison to *ndt80*Δ/*NDT80* (Fig 6A). These data indicate that the increased density of *ndt80*Δ/*NDT80* biofilm may be due, in part, to an increase in filamentous cells in the basal layer of the structure. We next asked if the hyperfilamentous phenotype for *ndt80*Δ/*NDT80* was limited to biofilm forming conditions or was a more general characteristic of this mutant. We, therefore, examined the colony morphology of *ndt80*Δ/*NDT80* on Spider medium at 37°C, standard filament-inducing conditions. As shown in Fig 6B, the *ndt80*Δ/*NDT80* strain showed dramatically increased central wrinkling relative to WT and, as with the biofilm density phenotype, these morphological changes were exquisitely dependent on both *TEC1* and *ROB1*. Thus, decreased *NDT80* gene dosage appears to trigger a compensatory response dependent on the *TEC1* and, potentially, a *TEC1-ROB1* pathway which leads to an increased filamentation. This increased filamentation, in turn, appears to create a dense basal layer within the biofilm.

### Quantitative characterization of the genetic interactions between biofilm TFs

The significant effects of single heterozygous, double heterozygous and homozygous deletion mutants on the biofilm network suggest that it is highly inter-dependent and integrated. Nobile et al. used ChIP and gene expression data to show that many of the TFs bind to each other’s promoters and, based on homozygous deletion mutant studies, affect expression [18]. Since these data were generated using mutants of single genes, the functional characteristics of the interactions between the TFs could not be assessed. In principle, the functional relationship between two TFs affecting a shared cellular process can be of three general types: 1) cooperative/synergistic; 2) independent; or 3) buffering/compensatory. To further explore the functional interactions of the TFs as a network, we used our set of biofilm density data from the all possible combinations of network TFs as the basis for a quantitative epistasis analysis.

Although there are a variety of quantitative models for genetic interaction analysis, the most widely applied approach is the multiplicative model [29, 30]. In the multiplicative model, a neutral or independent interaction is defined as a double mutant that displays a phenotype equal to the product of the effect of each of the individual mutants multiplied together [29]. For example, if two mutants each lead to a reduction in biofilm formation by 0.5 (effect of mutant normalized to WT = 1), then the double mutant would be expected to have a phenotype of 0.5 x 0.5 = 0.25. This phenotype is interpreted to mean that the effect of each individual mutation is manifest in the double mutant in a manner unaffected by the presence of the other mutation [29]. Double mutants with more severe phenotypes than the neutral phenotype imply that the two genes function inter-dependently or cooperatively [29]; double mutants with less severe phenotypes than the neutral phenotype suggest that the two genes function in the same pathway (diminishing returns effect) or compensate for one another [29]. These definitions have been used in the genome-wide scale *S*. *cerevisiae* projects [5, 6] and other yeast based epistasis analyses with double heterozygotes [31]. In addition, Mani et al. concluded that the multiplicative model had significant advantages over other models [29]. Therefore, we used the multiplicative model as the framework for our epistatic analysis.

**Fig 7:**
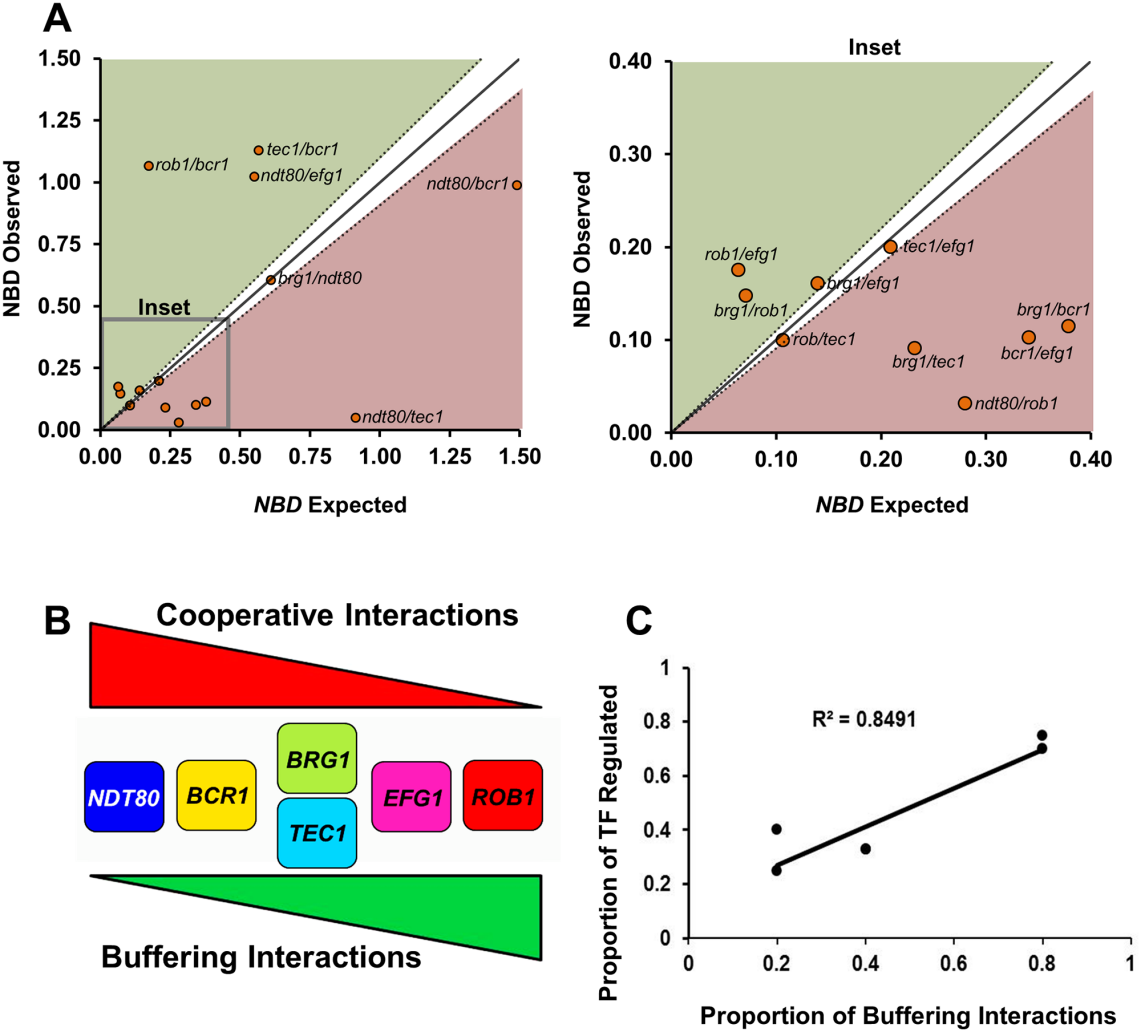
Epistasis analysis of the biofilm density for the biofilm TF network. (A) The nomarlized biofilm density (*NBD*) for each double heterozygote mutant was determined after 48 hr biofilm formation as described in Materials and Methods. The predicted NBD for each double heterozygous mutant was calculated by multiplying the *NBD* scores for each single mutant: NBD^*dh12*^ = NBD^*sh1*^ x NBD^*sh2*^, where *dh*^*12*^ is double heterozygote of single heterozygotes *sh*^*1*^ and *sh*^*2*^. The expected NBD^*dh12*^ is plotted against the observed NBD^*dh12*^. NBD^*dh12*^ scores that plot on the diagonal (solid line with estimated error indicated by dotted line) indicate no genetic interaction (independence) between the single mutants and correspond to Ɛ = 0; NBD^*dh12*^ below the diagonal have Ɛ <0 indicating an cooperative interactions; NBD^*dh12*^ above the diagonal have Ɛ > 0 indicating a buffering interaction. The plot on the left is an inset showing the details of the area indicated. The full data set for biofilm formation of all strains used to generate this data and calculations are provided in S2 Table. Strains with error that overlapped with the estimated error of the predicted NBD^*dh12*^ were considered to have no interaction. (B) Profile of cooperative and buffering interactions for each TF. (C) Correlation between number of buffering interactions and the number of network TF bound by that TF based on previously reported chromatin immunoprecipitation data. Pearson correlation coefficient is shown. Please note that *BRG1* and *TEC1* have identical numbers of buffering interactions and thus the data points overlap.

First, we normalized each single and double mutant biofilm density to WT (OD_600_ Mutant/OD_600_ WT). Second, we defined synergistic or cooperative interactions as mutants with normalized biofilm density significantly (Students t test) lower than neutrality and buffering or compensatory interactions as those with biofilm density higher than neutrality. As shown in Fig 7A, a plot of expected versus observed for each double mutant allows rapid identification of mutants with each type of interaction. Third, we calculated epistasis scores (Ɛ) for each mutant pair and these data are provided in S2 Table. Based on these calculations, we identified 6 cooperative and 5 buffering interactions along with 4 independent relationships. Each TF is part of at least one cooperative and at least one buffering interaction with a second network TF. Similarly, each TF has at least one other TF with which it functions in an independent fashion. Since both cooperative and independent interactions lead to reduced function relative to the individual mutants, only a small sub-set of gene pairs (33%) show the buffering effects that are frequently seen in networks of genes with overlapping functions [32].

The detailed transcriptional profiles and ChIP experiments performed by Nobile et al. [18] showed that the TFs of the biofilm network regulate themselves, other TFs, and biofilm-related targets or effector molecules. The overall function of each TF, therefore, represents a composite of these functions. We reasoned that the epistasis data may allow us to infer which of the functions predominate for a given TF. Because buffering interactions suggest linear relationships between genes [29], we further reasoned that buffering interactions between the biofilm TFs may represent the situation where one TF directly, or indirectly, regulates another TF within the overall network. Cooperative interactions, on the other hand, would most likely represent the interdependent convergence of two TF on either a set of common biofilm effector genes or, alternatively, on other TFs [29]. As shown in Fig 7B, the TF with the most cooperative interactions has the fewest buffering interactions (*NDT80*). Conversely, *ROB1* has the most buffering interactions and the fewest cooperative interactions. *BRG1* and *TEC1*, on the other hand, display an equal mix of cooperative and buffering interactions and, thus, do not appear to have a predominate mode of function within the network.

Next, we compared the proportion of buffering interactions for each TF to the number of network TFs whose expression is dependent on that TF based on the data from Nobile et al. [18]. The proportion of buffering interactions for a given TF correlated well the number of TFs whose expression it regulated (Fig 7C; R^2^ = 0.85). For example, *ROB1* has the highest proportion of buffering interactions and it regulates the expression of all but one of the other TFs in the network [18]. In contrast, Ndt80 regulates only one other TF and has the lowest number of buffering interactions. Furthermore, Rob1 directly regulates only a small proportion (2%) of the biofilm effectors while Ndt80 regulates the largest set of biofilm effectors of any TF in the network [18]. Based on these considerations, we propose, therefore, that Ndt80 predominately functions within the network through cooperative interactions with other TFs in the regulation of biofilm effectors. In contrast, Rob1 predominately regulates other TFs within network through linear or compensatory relationships. Between these two extremes, the other four TFs can be arrayed based on the relative proportion of cooperative and buffering interactions.

#### The biofilm TF network has properties of a small-world network

Many genetic networks display the property of robustness which is,”the ability to maintain performance in the face of perturbations [33]”. The biofilm TF network, on the other hand, is highly susceptible to genetic mutation and, therefore, is not robust, genetically. The topographical structure of a network can contribute to its ability to maintain robustness. Therefore, we calculated three standard network characteristics for the biofilm TF interaction network [34] to explore the relationship between network structure and function (Table 1). The degree (k) is the most fundamental characteristic and is the number of links a given node (TF) has to other nodes in the network. The degrees range from 3–5 with an average of 3.7 out of a possible 5, indicating the TFs are highly connected. The distance between nodes is expressed as the mean shortest path length (<l>) and represents the smallest number of links that are required to move from one node to another. Although only one TF (*BCR1*) is connected to all other network TFs, the average path length is quite short (<l>_net_ = 1.3). The most widely used measure of a network’s connectivity is the clustering coefficient (C_i_). C_i_ is a measure of the number of triangles that are contained within a network [34]. In other words, how many times are two nodes that are both connected to a common node also connected to each other. The C_i_ is a measure of a networks propensity to form clusters or groups: C_i_ = 1 indicates that all nodes connected to node i are also connected to themselves, while C_i_ = 0 indicates that none are connected to one another. Although only *EFG1* shows a C_i_ = 1, the other nodes are highly connected and the average C_i_ for the network is 0.74.

**Table 1:** Properties of biofilm TF network.

Transcription Factor	Degree (k)	Mean path Length(l)	Clustering Coefficient (*C*_*i*_)
**Efg1**	3	1.4	1.0
**Rob1**	4	1.4	0.75
**Ndt80**	4	1.2	0.75
**Bcr1**	5	1.0	0.60
**Brg1**	3	1.4	0.67
**Tec1**	3	1.4	0.67
**Network Average**	3.7	1.3	0.74

The combination of a short average path length (<*l*>) and a high clustering coefficient (*C*_*net*_) is characteristic of small-world networks as described by the Watts-Strogatz model [35] as well as others. Small world networks have highly connected components and are observed within networks that are random or scale-free [34,36]. If the overall structure is that of a random network, then the path length will be proportional to the logarithm of the number of nodes (*l* ~ *lnN*), whereas if the network is scale-free then the path length will be ultrashort and proportional to the double logarithm of *N* [34]. For this network, the <*l*> = 1.3 which is comparable to *ln*(6) = 1.7. Thus, the biofilm TF network properties are consistent with a random, small world network rather than a scale-free small world network. Furthermore, scale-free networks typically have hubs with significantly higher degrees relative to the other nodes. For the biofilm TF network, no TF shows a degree within ± 1 of the network average and, consequently, there is no dominant hub that clearly distinguishes itself from the others. As such, the structure of the biofilm TF network is more consistent with the uniform degree distribution typical of a random network.

The highly connected nature and short path lengths of small-world networks facilitate the rapid dissemination of information throughout the network, unless there are genetically isolated hubs within the overall structure of the network. Isolated hubs prevent deleterious perturbations from being transmitted to the entire network. Consequently, small-world networks can be characterized by high efficiency as opposed to high robustness. For example, Peng et al. have demonstrate that robustness and efficiency can represent conflicting properties of a network, i.e., networks with high efficiency are typically less robust and vice-versa [37]. The efficiency (*E*) of a network can be expressed by the following equation: *E* = 1/N(N-1) Σ1/*l*_*ij*_ where N is the total number of nodes and *l*_*ij*_ is the shortest path length between any given pair of nodes N_i_ and N_j_ [38]. Based on our data, the biofilm network is highly efficient with *E* = 0.82. The maximum value for *E* is 1 and occurs when all path lengths in a network are 1. As described by Netotea and Pongor [38], random networks without small-world characteristics have *E*~0.2 while small world networks have *E*> 0.5. Taken together, the topographical features of the biofilm network imply that it functions to efficiently relay information rather than to protect against genetic disruption.

#### The biofilm TF network contains feed-forward loops with superimposed positive feedback loops

**Fig 8:**
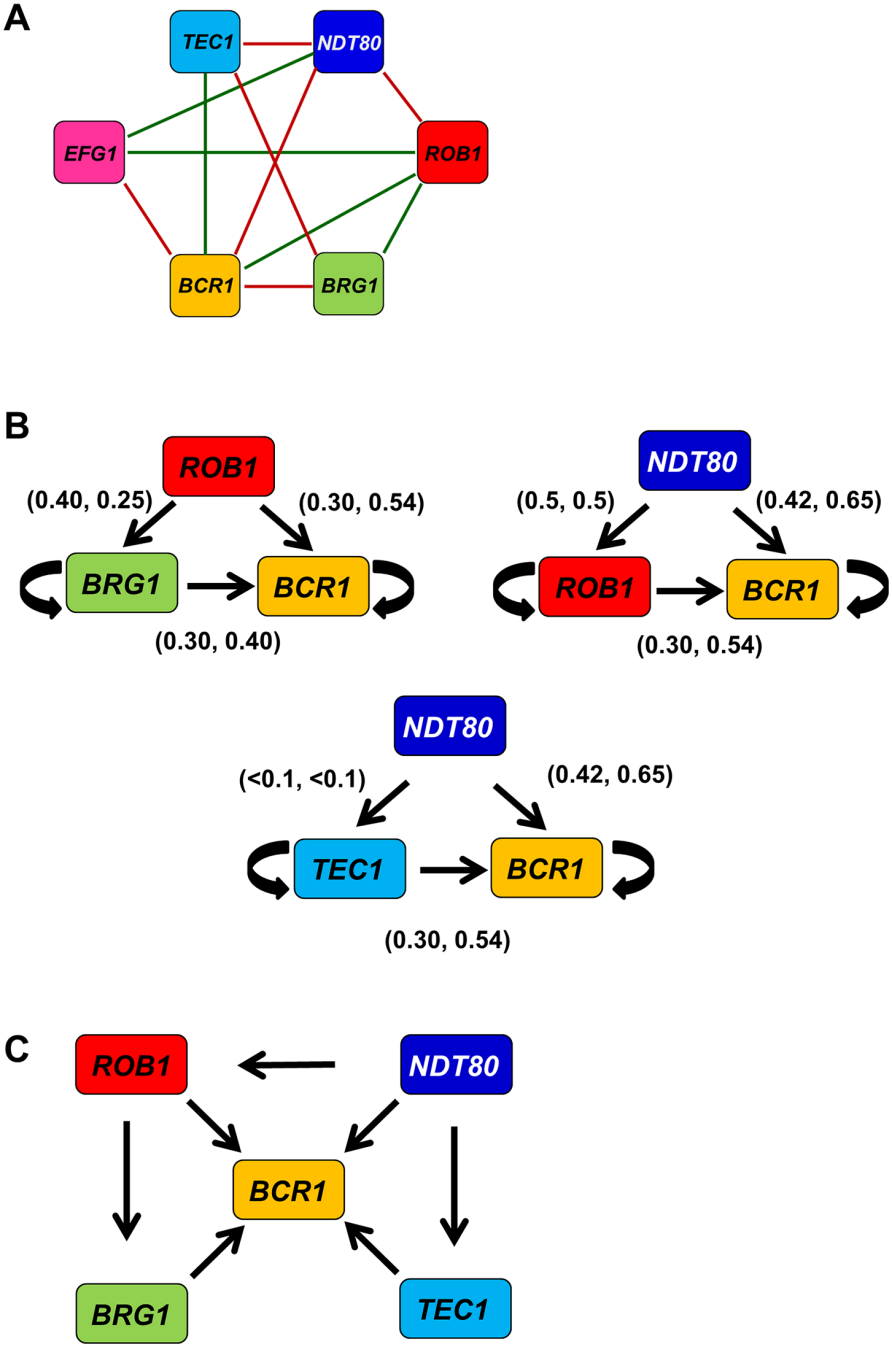
The biofilm TF network contains feedforward loops that regulate *BCR1* expression. (A) A summary of the phenotypic genetic interaction network for the biofilm TFs. (B) Three network modules that show both phenotypic interactions and downstream target expression changes consistent feedforward loops. The numbers in parenthesis over each arrow indicate the fold change in expression of the upstream and downstream TF (upstream, downstream) in the corresponding double mutant as compared to each single mutant. The arrows indicate that the double mutant has reduced expression of the TF at the head of the arrow. The circular arrrows indicate auto-regulation. All indicated expression changes are statistically significant (*P* < 0.05) and the primary data are provided in S2 Table. (C) The higher level module that results in the combination of the three feed-forward loops.

A feature that distinguishes a simple, random network from a small-world, random network is the presence of modules or motifs within the larger network structure [34, 35, 36]. One of the most prevalent sub-structures in TF networks is the triangular node loop [39]. Networks with high connectivity coefficients, by definition, have many of these triangular motifs; within the biofilm interaction network, there are seven such motifs (Fig 8A). Since each TF is regulated by multiple other network TFs, we were interested in identifying which of the three-node motifs represent feed-forward loops regulating the expression of network TFs. To do so, we used our set of TF expression data for each double mutant to determine if deletion of two TF alleles affected the expression of the downstream TF in a manner consistent with a feed-forward loop. Of the seven candidate loops, three showed effects on expression consistent with feed-forward circuits (Fig 8B). Specifically, each of the double mutants representing an edge of the putative feed-forward loop showed reduced expression of the downstream TF relative to either single mutant (>1.5 fold). Each of the three feed-forward loops regulates *BCR1* expression. The double mutants representing each edge show reduced expression of the downstream TF relative to the singe heterozygote of the upstream TF. This indicates that each target TF within the regulatory loop is subject to significant auto-regulation in addition to regulation by the upstream TF. As such, each of the three TF modules has properties of both feed-forward and positive feedback loops.

All three feed-forward modules share an edge and, therefore, can be arranged as higher order motif centered on *BCR1*. Rob1, Brg1, and Tec1 are known to regulate *BCR1* expression based on the analysis of the corresponding homozygous deletion mutants (Fig 8C). Deletion of *NDT80* does not affect *BCR1* expression but Ndt80 binds to the promoter of Bcr1, suggesting that it may play an ancillary role that is only manifest in the setting of another TF deletion. Thus, our expression data indicate that three of the putative feed-forward loops are likely to function as such, at least in part. Additionally, the data indicate that a high order directional network linking five of the six TFs to the regulation of *BCR1* expression can be constructed from the integration of genetic interaction data based on both functional (biofilm density) and gene expression readouts.

## Discussion

The *C*. *albicans* biofilm TF network is made up of a set of six genes whose homozygous deletion mutants have significantly decreased biofilm formation [18]. Consequently, the analysis of double homozygous deletions as an approach to understanding the inter-network genetic interactions of the TFs would be limited because the effect of a second homozygous mutation would be difficult to quantify. We hypothesized that the analysis of single and double heterozygous mutants and, thereby, simple and complex haploinsufficiency might provide information regarding the phenotypic interactions between the network TFs. As detailed above, this approach has provided additional insights into the function of specific TFs that were not accessible through the study of homozygous deletions mutants. Consequently, the complex haploinsufficiency-based approach offers complementary information that can add to our understanding of genetic networks in *C*. *albicans*.

One of the striking findings of our experiments is that all of the TF mutants showed simple haploinsufficiency as measured by biofilm density or confocal microscopy. This rate of haploinsufficiency is significantly higher than that observed for large collections of heterozygous *C*. *albicans* or *S*. *cerevisiae* mutants [22, 23]. Similarly, we observed that 10 of the possible 15 double heterozygous mutants showed decreased biofilm density relative to the single mutants and many of these showed phenotypes as severe as double homozygous mutants. Although many genetic networks buffer the cell against the deleterious effects of genetic mutations (the property of robustness, [40]), the biofilm TF network shows limited capacity to withstand genetic mutations.

Initially, this was somewhat surprising because Nobile et al found that the members of this network bound to other network TFs promoters implying that the expression of each TF may be controlled by multiple redundant contributions from other network TFs [18]. Additionally, the majority of the target genes TFs are also regulated by multiple network TFs [18]. The exquisite sensitivity of the function of the TF network to variation in gene dosage is, however, inconsistent with a robust network [33, 37, 38, 40]. Indeed, this network is quite fragile. It is important, however, to keep in mind that network robustness is not the only quality for which a network may be adapted. Specifically, networks with decreased robustness frequently display increased efficiency [37, 38], which is defined as the ability to rapidly transmit information throughout the network based on the properties of highly inter-connected nodes and short path lengths between linking each node to another node [38]. Such networks are examples of small world or Watts-Strogatz networks [35, 36]; this property is expressed as efficiency (*E*). As we have detailed above, the biofilm TF network appears to be best characterized as a small world network that displays high efficiency and low robustness. The Watts-Strogatz network structure has been proposed to best approximate the genetic network that regulates fat storage in yeast [41].

An example of the efficiency, as opposed to robustness, displayed by the biofilm TF network is the regulation of *TEC1* expression. Homozygous deletion of each network TF, with the exception of *NDT80*, leads to decreased *TEC1* expression (3- > 10-fold, [18]). Similarly, deletion of one allele of four TFs (*TEC1*, *EFG1*, *ROB1* & *BRG1*) reduce *TEC1* expression by at least 1.5-fold (Fig 2B). Thus, perturbation of the network at multiple nodes leads to reduced *TEC1* expression indicating that it is an important output of the TF network and that it is highly connected to the functional state of the network in general. Based on the observation that the *TEC1* heterozygote shows haploinsufficiency and a 2-fold reduction in *TEC1* expression, a significant portion of the biofilm deficits observed in the deletion mutants of network TFs could be explained by reduced *TEC1* expression. The importance of *TEC1* to the function of the network is also highlighted by the effect of rendering *TEC1* expression independent of the biofilm network. Under these conditions, the biofilm densities for the *BRG1* and *EFG1* heterozygotes are increased indicating that loss of network-mediated expression of *TEC1* contributes to the phenotypes of these mutants.

These observations can be contrasted with what would be expected if the biofilm network functioned in a robust manner [32]. *TEC1* appears to be both a component and an output of the biofilm network. As such, relatively modest changes in *TEC1* expression lead to significant changes in phenotype. If the biofilm network were functioning in a robust manner, then the expression of *TEC1* would be less susceptible to small changes in the function of other nodes and hence would be “buffered”. Instead, it appears that the structure and function of the biofilm TF network contributes to the ability of small perturbations at a number of nodes to be communicated through the network to regulate the expression of a functionally important member of the network.

We have focused on the network of TFs that regulate biofilm formation. This network is a sub-network within the larger biofilm genetic network containing both TFs and target genes [18, 20]. We were interested to explore whether the small-world properties exhibited by the TF portion of the network were shared by the overall network. Sorrell and Johnson have shown that the degree distribution of the overall network has a negative slope consistent with a scale-free network rather than the properties of a random network shown by the TF sub-network [42]. Importantly, one of the properties of scale-free networks is that their sub-networks can have different topological structure, e.g., randomness, [43]. In addition, both random and scale-free networks can have small world properties [34].

Sorrell and Johnson also reported that 3145 three-node modules exist within the biofilm TF-target network, indicating a high coefficient of connectivity [42]. Therefore, the overall structure is most consistent with a scale-free network with small-world properties. Scale-free small world networks have short path lengths that can be estimated by <*l*> = ln(ln *N*) where *N* = the number of nodes [34]. Application of this equation to the overall network estimates that the average path length is ~ 1.93, very similar to that of the biofilm TF network. Taken together, both the overall biofilm network and the local TF network show the high connectivity and short path length characteristic of a small world network. For the TF network, the small world property is in the context of a random network while the global network has features that appear more consistent with a scale-free network. One of the properties of scale-free networks is that their sub-networks can have different topological structure, e.g., randomness, while subnetworks in large random or exponential networks retain the same topology [43].

Therefore, the overall structure of the biofilm network is consistent with, not surprisingly, a set of TF hubs making up a sub-network embedded within the global biofilm network. This model is immediately recognizable from the work of Nobile et al. [18] and is not a novel idea. The data reported herein, however, allowed us to probe the question, “how do the hubs interact with one another?” The hubs of a given network can be sparsely connected to one another or highly connected [34, 40]. The former situation is referred to as an assortative relationship while the latter is a dissortative relationship. Dissortative networks in which highly connected hubs are isolated genetically from other hubs prevent the rapid, network-wide communication of deleterious perturbations. A widely cited example of a dissortative network is the yeast protein-proten interactome, although this view has been challenged [40, 44]. Small world networks (e.g., social networks) are examples of assortative relationships in that highly connected hubs are themselves interconnected [34].

The data reported herein and by Nobile et al. [18] indicate that the TF hubs of the biofilm network are highly connected and, therefore, represent an assortative small world, biological network. The structure and interactions of the biofilm TF network is such that full function of each node (TF) is required for normal biofilm formation. As such, the network represents a set of six “*and*” operators. In other words, Efg1 *and* Rob1 *and* Ndt80 *and* Tec1 *and* Bcr1 *and* Brg1 must all be at the proper level of function for a biofilm to form; small perturbations, e.g., reduction in gene expression by one-half, at any node leads to decreased function of the network. If this network were robust then it would, instead, be represented by a set of “*or*” operators. Specifically, biofilm formation will occur if Efg1 *or* Rob1…*or* Tec1 are fully functional. An “*or*” network insures that the function occurs under the widest possible set of conditions while an “*and*” network restricts the function to a very specific set conditions. Consequently, we propose that the functional interactions of the biofilm TFs and the topographical features of the biofilm network may contribute to a mechanism whereby *C*. *albicans* biofilm formation occurs only when specific genetic conditions are in place.

## Materials and methods

### Strains and plasmids

All heterozygous and double heterozygous deletion strains of *Candida albicans* were constructed from SN152 using auxotrophic markers of *LEU2* or *HIS1* which were flanked with homologous regions of 100-500bp for targeted integration [45, 46]. A complete list of strains, genotypes, and sources is provided in S1 Table. Deletion cassettes were amplified from the Homann et al. [16] homozygous deletion collection by PCR and using primers containing SbfI sites (primer sequences are provided in S3 Table). The amplification primers amplified both the *LEU2* and *HIS1* deletion cassettes. To differentiate between the two auxotrophic genes, the amplicons were digested with BglII which cuts in the *HIS1* gene but leaves the *LEU2* cassette intact. The *LEU2*-containing amplicons were cloned into pCR4TOPO (Invitrogen). The plasmid inserts were confirmed by sequencing. SbfI is used to excise the linear deletion cassette from pCR4-TOPO for transformation into *C*. *albicans* using standard lithium-acetate protocols. *LEU* transformants were screened for integration of the auxotrophic marker into the correct locus by PCR using primers internal to the deletion cassette and upstream of the integration site. Quantitative PCR was performed for each heterozygote and showed the expected reduction in copy number relative to the parental strain. Two independent isolates of each heterozygous deletion stain were used for biofilm determinations.

The *ndt80*Δ/*NDT80* mutant was complemented using the *NEU5L* pDUP3 shuttle vector method [46]. Briefly, the *NDT80* locus containing its endogenous promoter was amplified by PCR using primers containing flanking sequences homologous to pDUP3 (S3 Table) and introduced into the vector by gap-repair cloning in *S*. *cerevisiae*. The resulting pDUP3-*NDT80* construct was integrated into the *NEU5L* genomic site as previously described [46]. Complemented strains were selected on nourseothricin resistance (YPD + 400μg/mL nourseothricin).

Strains containing an allele of *TEC1* expressed from the *TDH3* promoter were generated by PCR amplification of the nourseothricin-marked *TDH3* promoter construct from plasmid pCJN542 (generous gift from Dr. Aaron Mitchell, [27]) with primers containing flanking sequence from the 5’ UTR upstream of the *TEC1* ATG and downstream of the ATG (S3 Table). Correct integration was confirmed by PCR.

### In vitro biofilm formation

*Candida* strains were grown overnight at 30°C with shaking in YPD liquid cultures. Cells were washed three times in PBS then re-suspended to an optical density (OD_600_) of 0.5 in biofilm-inducing media YETS (2.5% tryptone, 1.5% yeast extract, 1% sucrose, 25mM dibasic potassium phosphate, 4mM MgSO_4_ pH 7.1) [27]. 200μL of cells were allowed to adhere for 90 minutes at 37°C in 96 well, tissue culture treated, polystyrene plates. After 90 minutes, the media was removed and non-adhered cells washed off with PBS. The biofilm-inducing media was replaced and the cells incubated at 37°C, without shaking. At 48 hrs, the supernatant was aspirated off and the biofilms washed once with PBS. The biofilm density was measured by reading the optical density at 600nM [20, 21] using a SpectraMax plate reader. At least 3 replicate wells were analyzed for each strain for each experiment which was repeated independently 2–3 times.

### Gene expression during in vitro biofilm growth

The same procedure and media described for in vitro biofilm formation described were used with the following modifications. Larger scales were required to allow isolation of sufficiently high levels of RNA. As such, 4mL of cell suspension was incubated in a 6-well tissue culture plate to increase the yield of harvested cells. After 48 hours, the supernatant was removed and the biofilms washed twice with PBS to remove non-adherent cells. The biofilms were harvested by scraping and the RNA extracted using the Qiagen RNeasy Kit. The RNA samples were DNAse I treated and reverse transcribed into cDNA using the Biorad iScript kit. The quantitative real time PCR reactions contained Biorad IQ SybrGreen supermix and utilized primers described by Nobile et al. [18] (S3 Table). The qRT-PCR reaction was performed under the following conditions; 95°C-3’, [95°C-15s, 54.2°C-45s, 60°C-1’] 40 cycles, 60°C. Data analysis was performed using the ΔΔCt method, with actin (*ACT1*) as a reference. All reported data are the means of 3 biological replicates performed in technical triplicate with SEM. Statistical significance of differences from wild type was evaluated with a two-sided Student’s *t* test.

### Visualization of three-dimensional biofilm architecture

The 3D biofilm architecture of *C*. *albicans* single-species biofilms was examined using previously established protocols optimized for confocal biofilm imaging [47, 48]. *C*. *albicans* cells were stained with concanavalin A (ConA) lectin conjugated with tetramethylrhodamine (555/580 nm; Molecular Probes, Inc.) for 30 minutes with 40 μg/ml ConA. The confocal images of 48 hr biofilms were obtained with a multi-photon laser scanning microscope (SP8, Leica Microsystems) equipped with a 20X (1.0 numerical aperture) water immersion objective lens. The biofilm were excited at 840 nm and observed with a 598/628 non-descanned detector NDD3 for ConA. The confocal image series were generated by optical sectioning at each selected position and the Amira 5.4.1 software was used to create 3D renderings of the biofilm architecture [48].

### Quantitative epistasis analysis and calculation of clustering coefficients

The biofilm density for each mutant strain was normalized to the wild type reference strain and referred to as normalized biofilm density (NBD). Epistasis between two mutants (X and Y) was defined as *Ɛ* = NBD_XY_—NBD_X_NBD_Y_ where NBD_XY_ is the normalized biofilm density of the double mutant and NBD_X_ and NDB_Y_ are the normalized biofilm densities of the two heterozygotes. NBD_X_NBD_Y_ represents the expected NBD for the double mutant if there was no genetic interaction and, thus, *Ɛ* = 0 indicate that the two mutations function independently. As described by Hall et al., double mutants whose SEM range was completely outside of the standard relative error of the expected values were defined as having an epistatic interaction [31]. *Ɛ* > 1 indicates a buffering interaction and *Ɛ* < 1 indicates a cooperative interaction. *Ɛ* values for all interactions are provided in S2 Table. The clustering coefficient is *C*_*I*_ = 2n_I_/*k*(*k*-1) where n_I_ is the number of links connecting the *k* neighbors of node I to each other. *C*_*i*_ represents the total number of triangles centered on a given node divided by the total possible triangles if every one of the nodes neighbors were connected to one another. The efficiency (*E*) was calculated using the following equation: *E* = 1/N(N-1) Σ1/*l*_*ij*_ where N is the total number of nodes and *l*_*ij*_ is the shortest path length between any given pair of nodes N_i_ and N_j_ [38].

## Supporting information

S1 Table Strain table. List of all strains used in this article.
(DOCX)

S2 Table Biofilm formation and TF expression in single and double heterozygous strains. In vitro biofilm formation data, quantitative genetic interaction calculations, and reverse-transcriptase PCR expression levels of the six biofilm TFs for each mutant. The data are means of triplicates with SEM.
(XLSX)

S3 Table Table of primer sequences. List of primers used to create strains and measure TF expression levels. qRT-PCR primers were also used in the quantitation of TF copy number in heterozygous strains (data not shown).
(XLSX)

S1 Fig Normalized biofilm density for each series of double heterozygotes compared to the parental heterozygote. The data are graphical representations of the numerical data provided in S2 Table. The OD_600_ readings were normalized to WT to give a normalized biofilm density (NDB). The data are means of triplicates and the error bars represent SEM.
(TIF)

S2 Fig Confocal microscopy images of *ROB1*, *EFG1*, *BRG1* heterozygotes with SN250 wild type reference. Images are of 48 hr biofilms stained with Concanavalin A as described in materials and methods.
(TIF)

## References

[1] Köhler JR, Casadevall A, Perfect J. The spectrum of fungi that infects humans. Cold Spring Harb Perspect Med. 2014;5:a019273 doi: 10.1101/cshperspect.a019273 25367975 10.1101/cshperspect.a019273 PMC4292074

[2] Jabra-Rizk MA, Kong EF, Tsui C, Nguyen MH, Clancy CJ, Fidel PL Jr, Noverr M. Candida albicans pathogenesis: fitting within the host-microbe damage response framework. Infect Immun. 2016;84:2724–2739. doi: 10.1128/IAI.00469-16 27430274 10.1128/IAI.00469-16 PMC5038058

[3] Hernday AD, Noble SM, Mitrovich QM, Johnson AD. Genetics and molecular biology in Candida albicans. Methods Enzymol. 2010;470:737–758. doi: 10.1016/S0076-6879(10)70031-8 20946834 10.1016/S0076-6879(10)70031-8

[4] Kim J, Sudbery P. *Candida albicans*, a major human fungal pathogen. J Microbiol. 2011;49:171–177. doi: 10.1007/s12275-011-1064-7 21538235 10.1007/s12275-011-1064-7

[5] Tong AH, Lesage G, Bader GD, Ding H, Xu H, Xin X, et al Global mapping of the yeast genetic interaction network. Science. 2004;303:808–813. doi: 10.1126/science.1091317 14764870 10.1126/science.1091317

[6] Costanzo M, VanderSluis B, Koch EN, Baryshnikova A, Pons C, Tan G, et al A global genetic interaction network maps a wiring diagram of cellular function. Science. 2016;353:6306. 10.1126/science.aaf1420 PMC5661885 27708008

[7] Hickman MA, Zeng G, Forche A, Hirakawa MP, Abbey D, Harrison BD, et al The 'obligate diploid' *Candida albicans* forms mating-competent haploids. Nature. 2013;494:55–59. doi: 10.1038/nature11865 23364695 10.1038/nature11865 PMC3583542

[8] Bennett RJ. The parasexual lifestyle of *Candida albicans*. Curr Opin Microbiol. 2015;28:10–17. doi: 10.1016/j.mib.2015.06.017 26210747 10.1016/j.mib.2015.06.017 PMC4688137

[9] Vyas VK, Barrasa MI, Fink GR. A Candida albicans CRISPR system permits genetic engineering of essential genes and gene families. Sci Adv. 2015;1:e1500248 doi: 10.1126/sciadv.1500248 25977940 10.1126/sciadv.1500248 PMC4428347

[10] Huang MY, Mitchell AP. Marker recycling in Candida albicans through CRISPR-Cas9-induced marker xxcision. mSphere. 2017 3 15;2:e00050–17. doi: 10.1128/mSphere.00050-17 28317025 10.1128/mSphere.00050-17 PMC5352831

[11] Stearns T, Botstein D. Unlinked noncomplementation: isolation of new conditional-lethal mutations in each of the tubulin genes of *Saccharomyces cerevisiae*. Genetics. 1988;119(2):249–260. 3294100 10.1093/genetics/119.2.249 PMC1203409

[12] Haarer B, Viggiano S, Hibbs MA, Troyanskaya OG, Amberg DC. Modeling complex genetic interactions in a simple eukaryotic genome: actin displays a rich spectrum of complex haploinsufficiencies. Genes Dev. 2007;21:148–159. doi: 10.1101/gad.1477507 17167106 10.1101/gad.1477507 PMC1770898

[13] Bharucha N, Chabrier-Rosello Y, Xu T, Johnson C, Sobczynski S, Song Q, et al A large-scale complex haploinsufficiency-based genetic interaction screen in *Candida albicans*: analysis of the RAM network during morphogenesis. PLoS Genet. 2011;7:e1002058 doi: 10.1371/journal.pgen.1002058 22103005 10.1371/journal.pgen.1002058 PMC3084211

[14] Saputo S, Norman KL, Murante T, Horton BN, Diaz Jde L, DiDone L, et al Complex haploinsufficiency-based genetic analysis of the NDR/Lats Kinase Cbk1 provides insight into its multiple functions in *Candida albicans*. Genetics. 2016;203:1217–1233. doi: 10.1534/genetics.116.188029 27206715 10.1534/genetics.116.188029 PMC4937472

[15] Xu D, Jiang B, Ketela T, Lemieux S, Veillette K, Martel N, et al Genome-wide fitness test and mechanism-of-action studies of inhibitory compounds in *Candida albicans*. PLoS Pathog. 2007;3:e92 doi: 10.1371/journal.ppat.0030092 17604452 10.1371/journal.ppat.0030092 PMC1904411

[16] Homann OR, Dea J, Noble SM, Johnson AD. A phenotypic profile of the *Candida albicans* regulatory network. PLoS Genet. 2009;5:e1000783 doi: 10.1371/journal.pgen.1000783 20041210 10.1371/journal.pgen.1000783 PMC2790342

[17] Noble SM, French S, Kohn LA, Chen V, Johnson AD. Systematic screens of a *Candida albicans* homozygous deletion library decouple morphogenetic switching and pathogenicity. Nat Genet. 2010;42:590–598. doi: 10.1038/ng.605 20543849 10.1038/ng.605 PMC2893244

[18] Nobile CJ, Fox EP, Nett JE, Sorrells TR, Mitrovich QM, Hernday AD, et al A recently evolved transcriptional network controls biofilm development in *Candida albicans*. Cell. 2012;148:126–138. doi: 10.1016/j.cell.2011.10.048 22265407 10.1016/j.cell.2011.10.048 PMC3266547

[19] Taff HT, Nett JE, Andes DR. Comparative analysis of Candida biofilm quantitation assays. Med Mycol. 2012;50:214–218. doi: 10.3109/13693786.2011.580016 21539503 10.3109/13693786.2011.580016 PMC3251704

[20] Lohse MB, Gulati M, Valle Arevalo A, Fishburn A, Johnson AD, Nobile CJ. Assesment and optimization of Candida albicans in vitro biofilm assays. Antimicrob. Agents Chemother. 2017;61:e02749–16. doi: 10.1128/AAC.02749-16 28289028 10.1128/AAC.02749-16 PMC5404589

[21] Fox EP, Bui CK, Nett JE, Hartooni N, Mui MC, Andes DR, Nobile CJ, Johnson AD. An expanded regulatory network temporally controls Candida albicans biofilm formation. Mol Microbiol. 2015;96:1226–1239. doi: 10.1111/mmi.13002 25784162 10.1111/mmi.13002 PMC4464956

[22] Deutschbauer AM, Jaramillo DF, Proctor M, Kumm J, Hillenmeyer ME, Davis RW, et al Mechanisms of haploinsufficiency revealed by genome-wide profiling in yeast. Genetics. 2005;169:1915–1925. doi: 10.1534/genetics.104.036871 15716499 10.1534/genetics.104.036871 PMC1449596

[23] Norris M, Lovell S, Delneri D. Characterization and prediction of haploinsufficiency using systems-level gene properties in yeast. G3 (Bethesda). 2013;3:1965–1977. 24048642 10.1534/g3.113.008144 PMC3815059

[24] Springer M, Weissman JS, Kirschner MW. A general lack of compensation for gene dosage in yeast. Mol Syst Biol. 2010;6:368 doi: 10.1038/msb.2010.19 20461075 10.1038/msb.2010.19 PMC2890323

[25] Uhl MA, Biery M, Craig N, Johnson AD. Haploinsufficiency-based large-scale forward genetic analysis of filamentous growth in the diploid human fungal pathogen *C*.*albicans*. EMBO J. 2003;22:2668–2678. doi: 10.1093/emboj/cdg256 12773383 10.1093/emboj/cdg256 PMC156753

[26] Muzzey D, Sherlock G, Weissman JS. Extensive and coordinated control of allele-specific expression by both transcription and translation in *Candida albicans*. Genome Res. 2014;24:963–973. doi: 10.1101/gr.166322.113 24732588 10.1101/gr.166322.113 PMC4032860

[27] Nobile CJ, Andes DR, Nett JE, Smith FJ, Yue F, Phan QT, et al Critical role of Bcr1-dependent adhesins in *C*. *albicans* biofilm formation in vitro and in vivo. PLoS Pathog. 2006;2:e63 doi: 10.1371/journal.ppat.0020063 16839200 10.1371/journal.ppat.0020063 PMC1487173

[28] Finkel JS, Xu W, Huang D, Hill EM, Desai JV, Woolford CA, et al Portrait of *Candida albicans* adherence regulators. PLoS Pathog. 2012;8:e1002525 doi: 10.1371/journal.ppat.1002525 22359502 10.1371/journal.ppat.1002525 PMC3280983

[29] Mani R, St Onge RP, Hartman JL 4th, Giaever G, Roth FP. Defining genetic interaction. Proc Natl Acad Sci U S A. 2008 3 4;105:3461–3466. doi: 10.1073/pnas.0712255105 18305163 10.1073/pnas.0712255105 PMC2265146

[30] Segrè D, Deluna A, Church GM, Kishony R. Modular epistasis in yeast metabolism. Nat Genet. 2005;37:77–83. doi: 10.1038/ng1489 15592468 10.1038/ng1489

[31] Hall DW, Agan M, Pope SC. Fitness epistasis among 6 biosynthetic loci in the budding yeast Saccharomyces cerevisiae. J. Heredity 2010; 101(S1): S75–S84. 10.1093/jhered/esq007 20194517

[32] Patra B, Kon Y, Yadav G, Sevold AW, Frumkin JP, Vallabhajosyula RR, Hintze A, Østman B, Schossau J, Bhan A, Marzolf B, Tamashiro JK, Kaur A, Baliga NS, Grayhack EJ, Adami C, Galas DJ, Raval A, Phizicky EM, Ray A. A genome wide dosage suppressor network reveals genomic robustness. Nucleic Acids Res. 2017;45:255–270. doi: 10.1093/nar/gkw1148 27899637 10.1093/nar/gkw1148 PMC5224485

[33] Kitano H. Towards a theory of biological robustness. Mol. Sys. Biol. 2007;3:137. 10.1038/msb4100179 PMC2013924 17882156

[34] Barabási AL, Oltvai ZN. Network biology: understanding the cell's functional organization. Nat Rev Genet. 2004;5:101–113. doi: 10.1038/nrg1272 14735121 10.1038/nrg1272

[35] Watts DJ, Strogatz SH. Collective dynamics of ‘small world’ networks. Nature 393:440–442. doi: 10.1038/30918 9623998 10.1038/30918

[36] Wang XF, Chen G. Complex networks: small world, scale-free, and beyond. IEEE Circ. Syst. 2003; 6–20.

[37] Peng G-S, Tan S-Y, Wu J, Holme P. Trade-offs between robustness and small-world effect in complex networks. Sci. Rep. 2016; 6:37317 doi: 10.1038/srep37317 27853301 10.1038/srep37317 PMC5112524

[38] Netotea S, Pongor S. Evolution of robust and efficient system topologies. Cell. Immunol. 2006;244:80–83. doi: 10.1016/j.cellimm.2006.12.007 17433277 10.1016/j.cellimm.2006.12.007

[39] Yeger-Lotem E, Sattath S, Kashtan N, Itzkovitz S, Milo R, Pinter RY, Alon U, Margalit H. Network motifs in integrated cellular networks of transcription-regulation and protein-protein interaction. Proc Natl Acad Sci U S A. 2004;101:5934–5939. doi: 10.1073/pnas.0306752101 15079056 10.1073/pnas.0306752101 PMC395901

[40] Maslov S, Sneppen K. Specificity and stability in topology of protein networks. Science 2002;296:910–913. doi: 10.1126/science.1065103 11988575 10.1126/science.1065103

[41] Al-Anzi B, Arpp P, Gerges S, Ormerod C, Olsman, Zinn K. Experimental and computational analysis of a large protein network that controls fat storage reveals the design principles of a signaling network. PLoS Comp. Biol. 2015;11:31004264. 10.1371/journal.pcbi.1004264 PMC4447291 26020510

[42] Sorrells TR, Johnson AD. Making sense of transcription networks. Cell. 2015;161:714–723. doi: 10.1016/j.cell.2015.04.014 25957680 10.1016/j.cell.2015.04.014 PMC4531093

[43] Stumpf MPH, Wiuf C, May RM. Subnets of scale-free networks are not scale-free: sampling properties of networks. Proc. Nat. Acad. Sci. USA 2005;102:4221–4224. doi: 10.1073/pnas.0501179102 15767579 10.1073/pnas.0501179102 PMC555505

[44] Batada NN, Reguly T, Breitkreutz A, Boucher L, Breitkreutz B-J, Hurst LD, Tyers M. Stratus not altocumulus: a new view of the yeast protein interaction network. PLoS Biol. 2006;4:e317 doi: 10.1371/journal.pbio.0040317 16984220 10.1371/journal.pbio.0040317 PMC1569888

[45] Noble SM, Johnson AD. Strains and strategies for large-scale gene deletion studies of the diploid human fungal pathogen *Candida albicans*. Eukaryot Cell. 2005;4:298–309. doi: 10.1128/EC.4.2.298-309.2005 15701792 10.1128/EC.4.2.298-309.2005 PMC549318

[46] Gerami-Nejad M, Zacchi LF, McClellan M, Matter K, Berman J. Shuttle vectors for facile gap repair cloning and integration into a neutral locus in *Candida albicans*. Microbiology. 2013;159:565–579. doi: 10.1099/mic.0.064097-0 23306673 10.1099/mic.0.064097-0 PMC3709822

[47] Xiao J, Klein MI, Falsetta ML, Lu B, Delahunty CM, Yates JR 3rd, et al The exopolysaccharide matrix modulates the interaction between 3D architecture and virulence of a mixed-species oral biofilm. PLoS Pathog. 2012;8:e1002623 doi: 10.1371/journal.ppat.1002623 22496649 10.1371/journal.ppat.1002623 PMC3320608

[48] Koo H, Xiao J, Klein MI, Jeon JG. Exopolysaccharides produced by *Streptococcus mutans* glucosyltransferases modulate the establishment of microcolonies within multispecies biofilms. J Bacteriol. 2010;192:3024–3032. doi: 10.1128/JB.01649-09 20233920 10.1128/JB.01649-09 PMC2901689

