# Supplementary material for: Genetic analysis of the Candida albicans biofilm transcription factor network using simple and complex haploinsufficiency
Source: PLoS Genet. 2017 Aug 9;13(8):e1006948. doi: 10.1371/journal.pgen.1006948 (PMC5565191; doi:10.1371/journal.pgen.1006948)
Supplement: S1 Table — List of all strains used in this article. (DOCX) [file pgen.1006948.s001.docx]

| Table S1 *Candida albicans* strains | | |
| --- | --- | --- |
| Strain | Genotype | Source |
| SN152 | CAI4 with *iro1::IRO1/iro1::λimm434, his1 /his1, leu2/leu2, arg4/arg4* | (1) |
| SN250 | CAI4 with *iro1::IRO1/iro1::λimm434 his1Δ/his1Δ, leu2Δ::C.dubliniensis HIS1 /leu2Δ::C.maltosa LEU2, arg4/arg4* | (1) |
| *brg1Δ/Δ* | as SN152 with *brg1Δ::LEU2/brg1Δ::HIS1* | (2) |
| *ndt80Δ/Δ* | as SN152 with *ndt80Δ::LEU2/ndt80Δ::HIS1* | (2) |
| *rob1Δ/Δ* | as SN152 with *rob1Δ::LEU2/rob1Δ::HIS1* | (2) |
| *tec1Δ/Δ* | as SN152 with *tec1Δ::LEU2/tec1Δ::HIS1* | (2) |
| *bcr1Δ/Δ* | as SN152 with *bcr1Δ::LEU2/bcr1Δ::HIS1* | (2) |
| *efg1Δ/Δ* | as SN152 with *efg1Δ::LEU2/efg1Δ::HIS1* | (2) |
| *brg1Δ/BRG1* | as SN152 with *brg1Δ::HIS1/BRG1* | This study |
| *ndt80Δ/NDT80* | as SN152 with *ndt80Δ::HIS1/NDT80* | This study |
| *rob1Δ/ROB1* | as SN152 *with rob1Δ::LEU2/ROB1* | This study |
| *tec1Δ/TEC1* | as SN152 *with tec1Δ::HIS1/TEC1* | This study |
| *bcr1Δ/BCR1* | as SN152 with *bcr1Δ::HIS1/BCR1* | This study |
| *efg1Δ/EFG1* | as SN152 with *efg1Δ::HIS1/EFG1* | This study |
| *brg1Δ/ndt80Δ* | as SN152 with *brg1Δ::LEU2/BRG1, ndt80Δ::HIS1/NDT80* | This study |
| *brg1Δ/rob1Δ* | as SN152 with *brg1Δ::LEU2/BRG1, rob1Δ::HIS1/ROB1* | This study |
| *brg1Δ/tec1Δ* | as SN152 with *brg1Δ::LEU2/BRG1, tec1Δ::HIS1/TEC1* | This study |
| *brg1Δ/bcr1Δ* | as SN152 with *brg1Δ::LEU2/BRG1, bcr1Δ::HIS1/BCR1* | This study |
| *brg1Δ/efg1Δ* | as SN152 with *brg1Δ::LEU2/BRG1, efg1Δ::HIS1/EFG1* | This study |
| *ndt80Δ/rob1Δ* | as SN152 with *ndt80Δ::HIS1/NDT80, rob1Δ::LEU2/ROB1* | This study |
| *ndt80Δ/tec1Δ* | as SN152 with *ndt80Δ::HIS1/NDT80, tec1Δ::LEU2/TEC1* | This study |
| *ndt80Δ/bcr1Δ* | as SN152 with *ndt80Δ::HIS1/NDT80, bcr1Δ::LEU2/BCR1* | This study |
| *ndt80Δ/efg1Δ* | as SN152 with *ndt80Δ::LEU2/NDT80, efg1Δ::HIS1/EFG1* | This study |
| *rob1Δ/tec1Δ* | as SN152 with *rob1Δ::HIS1/ROB1, tec1Δ::LEU2/TEC1* | This study |
| *rob1Δ/bcr1Δ* | as SN152 with *rob1Δ::HIS1/ROB1, bcr1Δ::LEU2/BCR1* | This study |
| *rob1Δ/efg1Δ* | as SN152 with *rob1Δ::LEU2/ROB1, efg1Δ::HIS1/EFG1* | This study |
| *tec1Δ/bcr1Δ* | as SN152 with *tec1Δ::LEU2/TEC1, bcr1Δ::HIS1/BCR1* | This study |
| *tec1Δ/efg1Δ* | as SN152 with *tec1Δ::LEU2/TEC1, efg1Δ::HIS1/EFG1* | This study |
| *bcr1Δ/efg1Δ* | as SN152 with *bcr1Δ::LEU2/BCR1,*  *efg1Δ::HIS1/EFG1* | This study |
| p*TDH3-TEC1* | as SN250 with *TEC1*/*TEC1:: pAgTEF1-NAT1-AgTEF1UTR-TDH3pr-TEC1* | This study |
| p*TDH3-TEC1*  *ndt80Δ/NDT80* | as SN152 with *ndt80Δ::HIS1/NDT80, TEC1*/*TEC1::pAgTEF1-NAT1-AgTEF1UTR-TDH3pr-TEC1* | This study |
| p*TDH3-TEC1*  *ndt80Δ/Δ* | as SN152 with *ndt80Δ::LEU2/ndt80Δ::HIS1, TEC1*/*TEC1:: pAgTEF1-NAT1-AgTEF1UTR-TDH3pr-TEC1* | This study |
| p*TDH3-TEC1*  *tec1Δ/TEC1* | as SN152 with *tec1Δ::HIS1/ TEC1::pAgTEF1-NAT1-AgTEF1UTR-TDH3pr-TEC1* | This study |
| p*TDH3-TEC1*  *brg1Δ/BRG1* | as SN152 with *brg1Δ::HIS1/BRG1, TEC1*/*TEC1:: pAgTEF1-NAT1-AgTEF1UTR-TDH3pr-TEC1* | This study |
| p*TDH3-TEC1*  *rob1Δ/ROB1* | as SN152 with *rob1Δ::LEU2/rob1Δ::HIS1, TEC1*/*TEC1:: pAgTEF1-NAT1-AgTEF1UTR-TDH3pr-TEC1* | This study |
| p*TDH3-TEC1*  *efg1Δ/EFG1* | as SN152 with *efg1Δ::HIS1/EFG1, TEC1*/*TEC1:: pAgTEF1-NAT1-AgTEF1UTR-TDH3pr-TEC1* | This study |
| p*TDH3-TEC1*  *rob1Δ/efg1Δ* | as SN152 with *rob1Δ::LEU2/ROB1, efg1Δ::HIS1/EFG1, TEC1*/*TEC1:: pAgTEF1-NAT1-AgTEF1UTR-TDH3pr-TEC1* | This study |
| *ndt80Δ/NDT80::NDT80* | as SN152 with *ndt80Δ::HIS1/NDT80, NEUT5L/NEUT5L–NAT1–NDT80–NEUT5L* | This study |

1. Noble SM, Johnson AD. Strains and strategies for large-scale gene deletion studies of the diploid human fungal pathogen Candida albicans. Eukaryot Cell. 2005;4(2):298-309.

2. Homann OR, Dea J, Noble SM, Johnson AD. A phenotypic profile of the Candida albicans regulatory network. PLoS Genet. 2009;5(12):e1000783.
