# Supplementary figures and images for: Genetic analysis of the Candida albicans biofilm transcription factor network using simple and complex haploinsufficiency
Source: PLoS Genet. 2017 Aug 9;13(8):e1006948. doi: 10.1371/journal.pgen.1006948 (PMC5565191; doi:10.1371/journal.pgen.1006948)

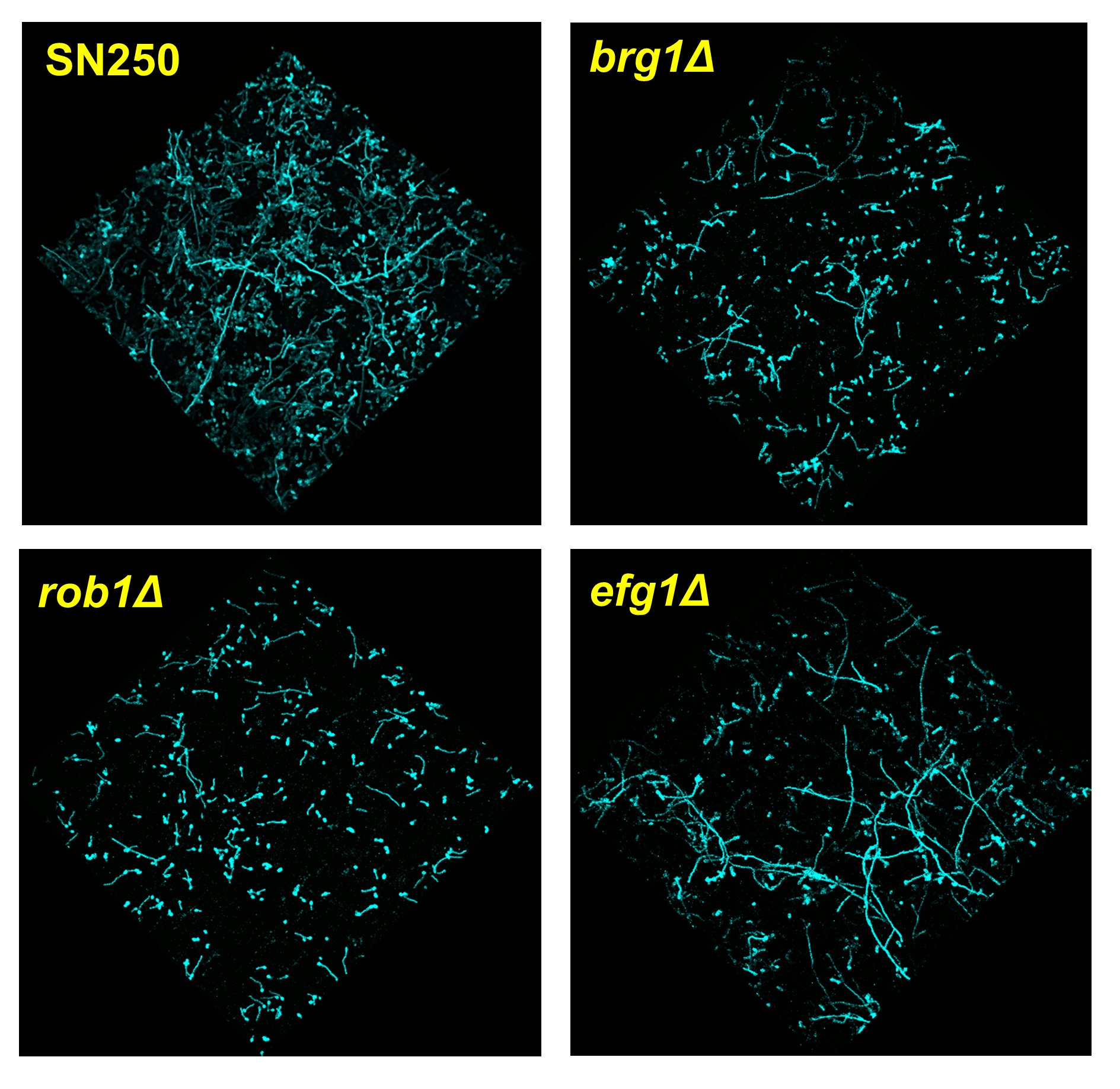

Supplement: S1 Fig — The data are graphical representations of the numerical data provided in S2 Table. The OD600 readings were normalized to WT to give a normalized biofilm density (NDB). The data are means of triplicates and the error bars represent SEM. (TIF) [file pgen.1006948.s004.tif]

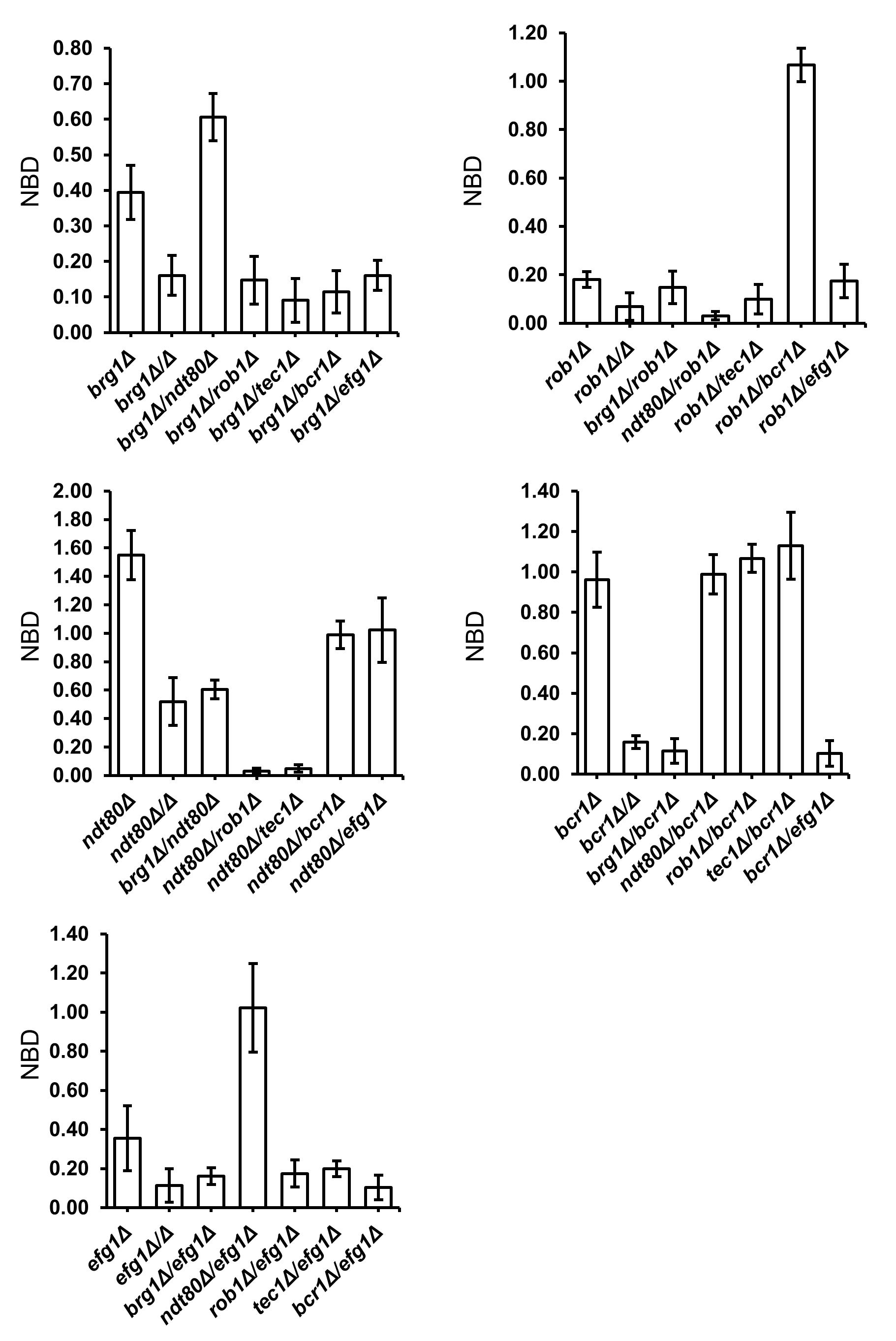

Supplement: S2 Fig — Images are of 48 hr biofilms stained with Concanavalin A as described in materials and methods. (TIF) [file pgen.1006948.s005.tif]
